# Growth hormone-releasing hormone agonist attenuates vascular calcification in diabetic db/db mice

**DOI:** 10.3389/fcvm.2023.1102525

**Published:** 2023-01-18

**Authors:** Hao-Lin Ren, Ruiping Cai, Ruize Xue, Yaoxia Zhang, Qian Xu, Xianyang Zhang, RenZhi Cai, Wei Sha, Andrew V. Schally, Ming-Sheng Zhou

**Affiliations:** ^1^Department of Radiology, The First Affiliated Hospital, Dalian Medical University, Dalian, China; ^2^Science and Research Center, Shenyang Medical College, Shenyang, China; ^3^Department of Physiology, Shenyang Medical College, Shenyang, China; ^4^Veterans Affairs Medical Center, Endocrine, Polypeptide and Cancer Institute, Miami, FL, United States; ^5^Miami Veterans Affairs Medical Center, South Florida VA Foundation for Research and Education, Miami, FL, United States; ^6^Divisions of Medical/Oncology and Endocrinology, Department of Pathology, Medicine, Miller School of Medicine, University of Miami, Miami, FL, United States; ^7^Sylvester Comprehensive Cancer Center, Miller School of Medicine, University of Miami, Miami, FL, United States

**Keywords:** vascular calcification, growth hormone-releasing hormone, diabetes, oxidative stress, vascular injury

## Abstract

**Introduction:**

Vascular calcification (VC) is an independent risk factor for cardiovascular diseases. VC increases mortality of all-causes. VC is one of most common cardiovascular complications in type II diabetes. So far, no therapy has been proven to be effective in treatment of clinical VC. The present study investigated the therapeutic effects of MR409, an agonistic analog of growth hormone-releasing hormone (GHRH-A), on VC in diabetic db/db mice.

**Method and result:**

Diabetic mice were injected with MR409 subcutaneously every day for 8 weeks. Long-term treatment with MR409 improved serum lipid profile and endothelium-dependent relaxation to acetylcholine, and reduced vascular structural injury in diabetic mice without affecting serum growth hormone level. Echocardiography showed that calcium plaques present in heart valve of diabetic mice disappeared in diabetic mice after treatment with MR409. MR409 inhibited vascular calcium deposition associated with a marked reduction in the expressions of osteogenic-regulated alkaline phosphatase (ALP) and transcription osteogenic marker gene Runx2 in diabetic mice. MR409 also inhibited vascular reactive oxygen species (ROS) generation and upregulated the expressions of anti-calcifying protein Klotho in diabetic mice.

**Discussion:**

Our results demonstrate that GHRH-A MR409 can effectively attenuate VC and heart valve calcification, and protect against endothelial dysfunction and vascular injury in diabetic mice without significantly affecting pituitary-growth hormone axis. The mechanisms may involve upregulation of anti-calcifying protein Klotho and reduction in vascular ROS and the expression of redox sensitive osteogenic genes Runx2 and ALP. GHRH-A may represent a new pharmacological strategy for treatment of VC and diabetics associated cardiovascular complications.

## Introduction

Vascular calcification (VC) is a pathological condition, which is characterized by an abnormal deposition of calcium-phosphate in the vascular system (1). The arterial medial calcification is the most common type of VC, which is frequently found in the patients with type II diabetes, atherosclerosis and end-stage of renal diseases (ESRDs) (2). It has been reported that the prevalence of VC is as high as 41.5% in patients with type II diabetes, and 27% in the patients with ESRDs (3). The calcification in the arteries increases arterial stiffness and reduces the elasticity and compliance of the artery wall, which may increase pulse pressure, heart afterload, deteriorate peripheral tissue perfusion and thrombus formation (4). As the consequence, VC increases the incidence of thrombosis, stroke, coronary ischemic disease, and plaque rupture. VC is considered an independent risk factor for cardiovascular morbidity and mortality (5, 6).

The mechanisms of VCs are not fully understood. Like the bone remodeling, VC is an active cellular process that is regulated by various bone-related proteins, such as alkaline phosphatase (ALP), osteocalcin, osteopontin, Run-related transcription factor 2 (Runx2), and matrix vesicles (7). Some agents, such as bisphosphonate, pyrophosphate analogs, have been tested to be effective in the prevention or regression of VC in the experimental animal models. However, to date, no effective agents have been found for the treatment or regression of VC associated with human diseases (8). Therefore, there is an urgent need to develop new strategies to prevent or treat VC.

Growth hormone-releasing hormone (GHRH) is a hypothalamic neuropeptide, which binds to the G-protein coupled GHRH receptor and stimulates the secretion and synthesis of growth hormone (GH) in the pituitary gland (9). In addition to its neuroendocrine action, GHRH and its receptors are expressed in different peripheral tissues and cell types, such as cardiomyocytes, vascular smooth muscle cells, neural cells, ocular tissue and pancreas (10–13). The extrahypothalamic GHRH can regulate many cellular functions, such as cell proliferation, differentiation and survival, in various peripheral cells and organs (14, 15). In past few decades, many potent GHRH agonists has been synthesized in our laboratory. These synthetic analogs of GHRH are more stable and potent when compared with native GHRH (14). It has been shown that GHRH analog MR409 can exert therapeutic effects on experimental acute myocardial infarction, heart failure and diabetic retinopathy (11, 16, 17). More recently, Shen et al. (18) reported that MR409 can suppress vascular smooth muscle calcification *in vitro* and VC in osteoprotegerin KO mice *in vivo* by blocking reactive oxygen species (ROS)/nuclear transcription factor (NF)*k*B pathway. However, osteoprotegerin KO mice is not an animal model that mimics VC in human diseases. In the present study, we investigated the therapeutic effects of MR409 on VC in diabetic db/db mice.

## Materials and methods

### Animal protocols

Ten-week-old male homozygous db/db mice on a C57BL/6 background were purchased from Model Animal Research Center of Nanjing University (MARC, Nanjing, China) and housed in an animal facility at 23 ± 2°C with a 12-h light/dark cycle, C57BL/6 mice were used as a wild type (WT) control. We used 10-week-old male mice in this study, because it has been reported that sex hormones, especially estrogen, may affect VC (19), and db/db mice can develop VC about 20-week-old (the age of mice at sacrifice) (20). All animal procedures were approved by the Institute Animal Use and Care Committee of Shenyang Medical College and complies with the Guideline of the Institute Care and Use of Laboratory Animals of Shenyang Medical College. After 2 weeks of adaptation, the mice were randomly divided into 3 groups and fed a regular mouse diet, and received one of the following treatments for 8 weeks: Control group (WT): C57BL/6 mice were injected subcutaneously with vehicle solution [10% (vol/vol) propylene glycol] every day; diabetic db/db group (db/db): db/db mice received a subcutaneous injection of vehicle solution every day; db/db mice in MR409 treatment group (MR409) were administrated subcutaneously with MR409 at a dose of 15 μg/day/mouse (dissolved in 10% propylene glycol). We have shown that MR409 at a dose of 10 μg/day/mouse has therapeutic effects on experimental stroke and STZ-induced diabetic mouse models (10, 12), we used MR409 at a dose of 15 μg/day/mouse, because db/db mice have more body weight than regular mice. The number of experimental animals was based on the statistical power analysis. MR409 was synthesized by our laboratory (12, 14). At the end of the study, the mice were fasted overnight, blood was taken from the tail arteries to determine fasting blood level of glucose with an automatic blood glucose meter (Roche Accu-CHEK Active, Mannheim, Germany). After insulin tolerance test was performed, the mice were euthanized with 100 mg/kg ketamine and 20 mg/kg xylazine cocktail, and blood was withdraw through left ventricular puncture. The heart and aorta were harvested.

### Insulin tolerance test

The mice were fasted for 4 h, insulin tolerance test was performed 2 days before the animals were sacrificed. Briefly, the mice were injected intraperitoneally with insulin solution (1 U insulin/kg body weight). Blood glucose levels were measured at 0 (before insulin administration), 20, 40, 60, 120, and 180 min after insulin injection using an automatic blood glucose meter (Roche Accu-CHEK Active, Mannheim, Germany).

### Histological analysis

Thoracic aorta was isolated and cut into 3 mm aortic ring, which was fixed in 4% paraformaldehyde buffered solution and routinely processed for paraffin sections. The slices of 4 μm thickness was cut and mounted in the slides. The slides were stained with hematoxylin eosin (HE, Sigma Aldrich, St. Louis, MO) to evaluate the thickness of aortic wall and morphological alteration. At least four images per stained sections were examined and photographed using a Leica DM4B fluorescence microscope (Leica Microsystems Inc., Mannheim, Germany). The thickness of aortic wall (from intima to media) was measured by ImageJ software (NIH Bethesda, MD), the average thickness of aortic wall was calculated. The separated sections were stained with Masson trichrome to evaluate vascular fibrosis, the positive stained area of collagen was quantified with ImageJ software, the percentage of positive stained collagen area with total stained area was calculated. The examination of histological samples was conducted in a blinded manner, the reviewers were not aware of the groups to which mice belonged.

### Vascular reactivity

Endothelium-dependent relaxation to acetylcholine was determined by an organ chamber bath as described previously (21). Briefly, isolated thoracic aorta was cut into 3 mm aortic rings. The aortic rings were mounted on the wires connected to pressure transducer in organ chamber bath system (DMT Inc., Denmark). The rings were equilibrated under resting tension of 1 g for 1 h, then contracted twice with PSS solution containing 60 mmol/L KCL. The rings were precontracted with about 30 nmol/L of norepinephrine to reach 70% of the maximum contractile force, then cumulative concentration of acetylcholine (10^–9^∼10^–5^ molar/L) was added to the organ bath. Maximal relaxation to acetylcholine (Emax) and the concentration of acetylcholine required for a half-maximal relaxation (ED50) were calculated using a best fit of a logistic sigmoid concentration-response curve.

### Assessment of heart valve and aortic calcification with echocardiography

At the end of an 8-week treatment, the mice were subjected to ultrasound examination for determination of aortic calcification and heart valve calcification, using a high-resolution Doppler image system for small animal (Esote Medical Equipment Inco., Shenzhen, China). The mice were anesthetized with the inhalation of 1% isoflurane, and placed on a platform and heated warm to maintain body temperature. The heart valves and aortic arches were examined and imaged with B-Mode or 2D mode cursor (30 MHz) in the parasternal short-axis view with a depth setting of 2 cm. The sample volume cursor was placed at the aortic root with angle correction (37°–60°). The heart valves and aortic calcification were determined by a significant change in ultrasound density. The areas of calcification spots or plaques were quantified by using ImageJ software.

### Alizarin red and Von Kossa calcium staining in mouse aortas

Alizarin red (AR) and Von Kossa (VK) were used for calcium staining of the mouse aortas. A segment of aortic arch was fixed in 10% formalin for 24 h, then dehydrated and embedded in paraffin. The section of 5-μm-thickness was cut and deparaffined. For AR staining, the slides were incubated with AR solution for 2 min (Solarbio Life Science, Beijing, China). After a quick wash in distilled water, the slides were placed under ultraviolet light until calcium phosphate turned black, followed by a counterstaining with eosin. For VK staining, deparaffined sections were stained with Von Kossa silver kits (Solarbio Life Science, Beijing, China) for 2 min. After washing with distilled water, the sections were rapidly differentiated and the nuclei stained with hematoxylin solution. Images were taken using microscope (Leica, Germany) and analyzed with ImageJ Software.

### Oil red O staining and ALP staining

Fresh aortic root was frozen and embedded in OCT compound and sliced into the section of 10 μm thickness by a microtome-cryostat (Leica, Germany). The sections were briefly rinsed with distilled water and immersed in 60% isopropanol for 30 s, then incubated in oil red O working solution (Solarbio Life Science, Beijing, China) for 15 min. The sections were then de-stained with 60% isopropanol, and counterstained with hematoxylin solution. Images were captured using Leica light microscope, and the percentage of positive staining area was quantified using ImageJ software. ALP staining in aortic section was performed with ALP staining kit following manufacturer’s instructions. Ten μm sections were cut, matrix solution was added to slices, and the tissue section incubated for 15 min at 37°C in dark environment. Excessive matrix solution was removed and cobalt nitrate dye added immediately and dyed for 5 min. Vulcanized solution was added and tissue sections incubated for 30 s. The slices were redyed with nuclear solid red dye for 30 s. The slices were shaken to dryness and examined by microscope.

### Immunohistochemistry

Paraffin-embedded aorta was cut into 4 μm thick sections. The slides were incubated with sodium citrate buffer for 10 min in a pressure cooker for antigen retrieval. After the incubation with blocking solution of 5% goat serum in TBST at room temperature for 10 min, the sections were incubated with primary antibodies against Runx2 (1:50 diluted with TBST buffer, SC-101145, Satan Cruz Biotech., USA) or Klotho (1:50 diluted with TBST buffer, ab181373, Abcam, UK) overnight at 4°C followed by the incubation with appropriate secondary antibodies conjugated with biotin for 15 min at room temperature. The sections were then incubated with HRP labeled streptavidin for 15 min at room temperature and stained with DAB solution. The nuclei were counterstained with hematoxylin solution. The images were acquired by a Leica DM4B fluorescence microscope and analyzed with ImageJ software. The results were expressed as a percentage of positive stained areas with total selected areas.

### Western blot

The whole aortas (from aortic arch to the bifurcation of abdominal aorta at femoral arteries) were lysed with RIPA lysis buffer containing a complete protease inhibitor cocktail. Protein concentrations were determined using a BCA protein assay kit (Beyotime Biotechnology, Shanghai, China). Protein samples (30 μg) were fractionated with SDS-PAGE. After electrophoretic transfer to a PVDF membrane (0.45 μm, IPVH00010, Millipore), the membranes were incubated with primary antibody against Klotho (1:1000 dilution, ab181373, Abcam) at 4°C overnight followed by the incubation with horseradish peroxidase-conjugated secondary antibody (1:5,000 dilution using blocking solution) for 2 h at room temperature, an antibody against β-actin (WH121414, ABclonal) as an internal control. The luminal chemiluminescence signals were detected with an Aplegen Omega Lum G Gel Documentation System (Aplegen Inc., Pleasanton). The band density was measured and quantified using ImageJ software.

### Determination of serum alkaline phosphatase (ALP) activity and growth hormone (GH)

Blood was centrifuged at 5000 rpm for 10 min at 4°C. Serum ALP activity was measured using ALP assay kit following the manufacturer’s instructions (Solebo Technology Co., Ltd). ALP assay kit is a p-nitrophenyl phosphate (pNPP) assay, ALP catalyzes pNPP (a phosphatase substrate) to pNP, which turns yellow at absorbance of 405 nm, which was measured using Microplate Absorbance Reader (Molecular Devices) for 15 min. One ALP unit is defined as hydrolyzation of 1 μmole of pNPP per minute at pH 9.8 at 37°C. QuantiChrom cholesterol assay kit (Coibo Bio. Inco., Shanghai, China). QuantiChrome triglyceride assay kit (Coibo Bio Inco., Shanghai, China) was used to determine serum concentration of total cholesterol, total triglyceride, respectively. Serum GH concentration was determined by mouse growth hormone enzyme-linked immunosorbent assay (ELISA) kit according to manufacturer’s instructions (Cusa Bio. Inco., Wuhan, China).

### Determination of superoxide (O_2_^–^) anion formation in aorta *in situ*

Aortic O_2_^–^ formation was determined by oxidative fluorescent dye hydroethidine (DHE, Sigma-Aldrich, St. Louis, MO) as described (21). In brief, thoracic aorta were embedded in OCT compound and snap-frozen in the liquid nitrogen. Sections of 5 μm thickness were cut and incubated with 2 μmol/L DHE in HEPES buffer for 30 min at 37°C in a humidified chamber. The images were acquired within 30 min of DHE staining, using a fluorescence microscope (Leica Microsystems Inc., Mannheim, Germany), and the average fluorescent intensities were quantitated and expressed as percentage of control.

### Statistical analysis

Statistical analysis was performed by SPSS statistical software (SPSS Inc, Chicago, IL). Data were presented as mean ± standard derivation (SD). Statistical analysis was performed by one-way or two-way ANOVA followed by the Student-Newman-Keuls test. *P* < 0.05 was considered statistically significant.

## Results

### Long-term treatment with GHRH-A MR409 reduced plasma lipid profile and vascular injury in diabetic db/db mice

**FIGURE 1:**
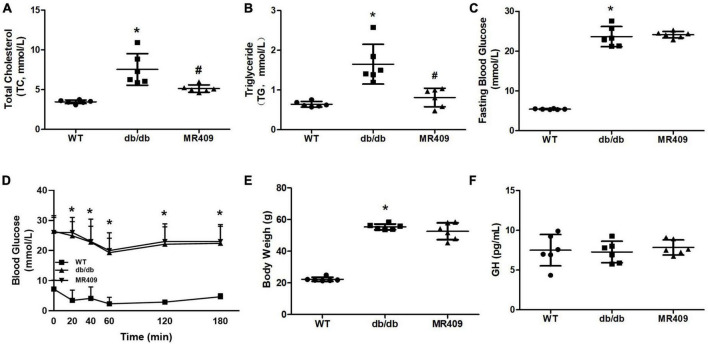
Effects of MR409 on plasma levels of total cholesterol (Ch, **A**) and total triglyceride (TG, **B**), fasting blood glucose **(C)**, insulin tolerance **(D)**, body weight **(E)** and serum level of growth hormone (GH, **F**) in diabetic db/db mice. Data is expressed mean ± SD, *n* = 6, *p < 0.05 diabetic mice vs. WT mice, #*p* < 0.05 MR409 treated mice vs. diabetic mice.

**FIGURE 2:**
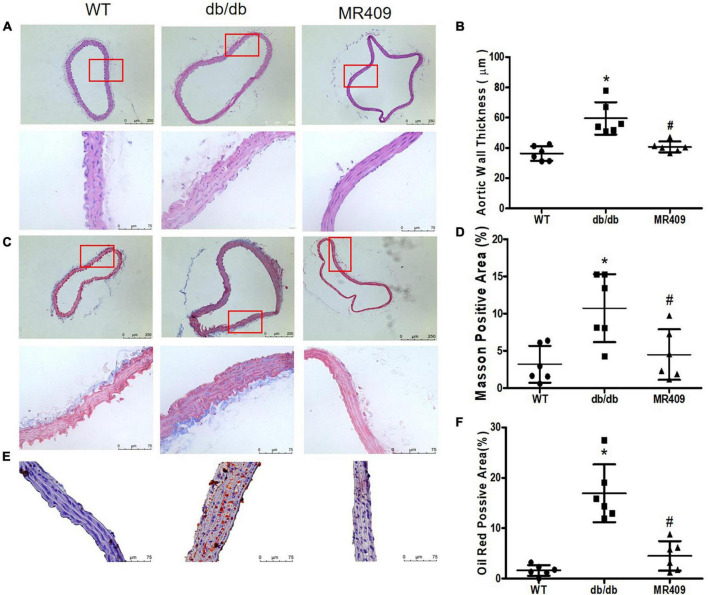
Treatment with MR409 improves vascular morphological injury in diabetic db/db mice. The representative images of cross section of aortic wall stained with hematoxylin and eosin for aortic wall thickness with a low magnification in up pane and a high magnification in low pane **(A)**, Masson trichrome for collagen content with a low magnification in up pane and a high magnification in low pane **(C)** and oil O staining for lipid deposition with high magnification **(E)**. The quantitation of aortic wall thickness **(B)**, positive collagen staining areas **(D)** and lipid deposition **(F)** in the aorta. *N* = 6, *p < 0.05 diabetic mice vs. WT mice, #*p* < 0.05 MR409 treated mice vs. diabetic mice.

Db/db mice at 20-week-old exhibited significant increases in fasting plasma level of total cholesterol, triglyceride and fasting blood level of glucose, impaired insulin tolerance and body weight, treatment with MR409 for 8 weeks lowered plasma lipid profile but did not significantly affect fasting blood glucose, insulin tolerance and body weight in db/db mice (Figures 1A–E). There was no significant difference in plasma level of GH between WT and diabetic db/db mice, and treatment with MR409 did not significantly affect plasma level of GH in diabetic mice (Figure 1F). HE staining showed that the thickness of aortic wall in diabetic mice significantly increased, which was prevented by MR409 treatment (Figures 2A, B). Masson trichrome staining revealed that diabetic mice had more positive staining area of aortic collagen and loosened elastic fiber structure, as compared with WT mice. MR409 treatment reduced aortic fibrosis and protected elastic fiber structure intact in diabetic db/db mice (Figures 2C, D). Lipid deposition was increased in the aorta of db/db mice, as showed by oil O staining, MR409 treatment significantly reduced aortic lipid deposition in db/db mice (Figures 2E, F).

### MR409 improved endothelium-dependent relaxation and reduced aortic ROS production in diabetic db/db mice

**FIGURE 3:**
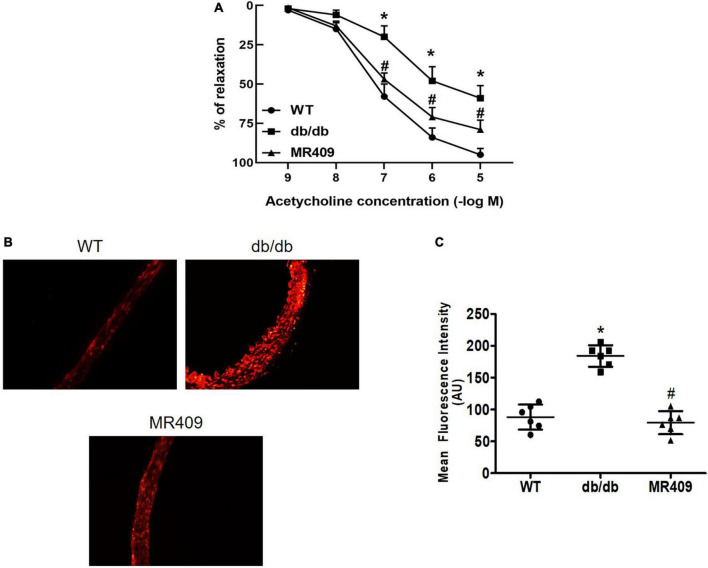
Treatment with MR409 improves endothelium-dependent relaxation to acetylcholine **(A)** and reduced reactive oxygen species (ROS) production in the aorta of diabetic db/db mice. **(B)** The representative images of oxidative fluorescence intensities in the aorta, assessed by DHE fluorescence staining, **(C)** the quantification of average oxidative fluorescence intensities. *N* = 6, *p < 0.05 diabetic mice vs. WT mice, #*p* < 0.05 MR409 treated mice vs. diabetic mice.

Endothelium-dependent relaxation to acetylcholine was significantly impaired in diabetic mice, MR409 treatment significantly improved acetylcholine-induced relaxation (Figure 3A). We determined aortic ROS production *in situ* by DHE oxidative fluorescence staining. As shown in Figures 3B, C, oxidative fluorescence intensity significantly increased in diabetic db/db mice, which was prevented by MR409 treatment.

### MR409 attenuated vascular calcium deposition and the protein expressions of osteogenic-regulated molecules in diabetic db/db mice

**FIGURE 4:**
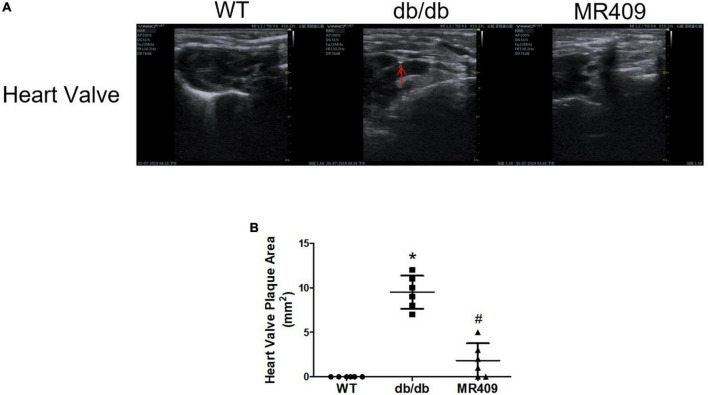
Treatment with MR409 attenuates heart valve calcification **(A)** in diabetic db/db mice. Heart valve calcification was detected with high-resolution Doppler image system for small animal. Representative images of heart valve **(A)** calcification, the quantitation of heart valve **(B)** calcification. *N* = 6, *p < 0.05 diabetic mice vs. WT mice, #*p* < 0.05 MR409 treated mice vs. diabetic mice.

**FIGURE 5:**
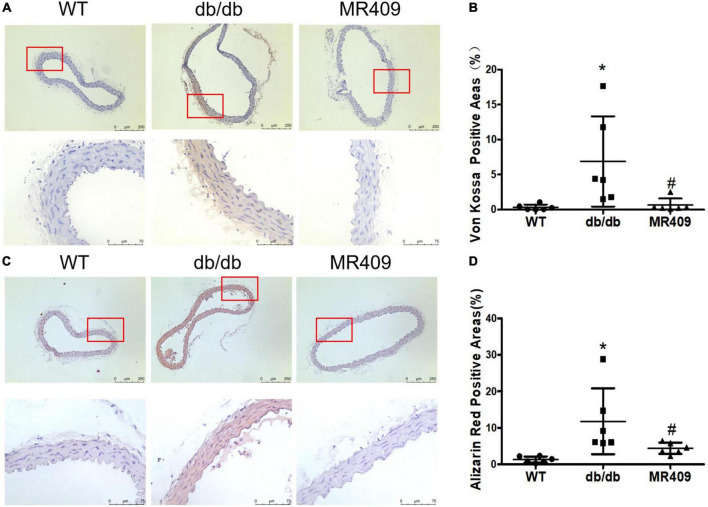
Treatment with MR409 attenuates calcium deposition in aortic root section of diabetic db/db mice. The representative images of calcium staining in the aorta by Von Kossa with a low magnification in up pane and a high magnification in low pane (brown, **A**) and alizarin red staining with a low magnification in up pane and a high magnification in low pane (dark red, **C**), the quantitation of positive calcium staining areas with Von Kossa **(B)** and alizarin red **(D)**. *N* = 6, *p < 0.05 diabetic mice vs. WT mice, #*p* < 0.05 MR409 treated mice vs. diabetic mice.

**FIGURE 6:**
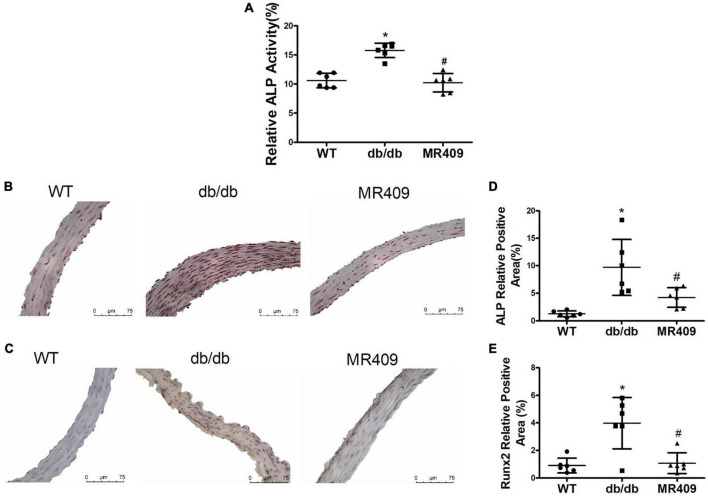
Treatment with MR409 reduces serum alkaline phosphatase (ALP) activity **(A)**, the expression of ALP **(B,D)** and Runx2 **(C,E)** in the aorta of diabetic db/db mice. The representative images of ALP **(B)** and Runx2 **(C)**, were determined by immunohistochemistry, the quantitation of positive staining areas of ALP **(D)** and Runx2 **(E)**. *N* = 6, *p < 0.05 diabetic mice vs. WT mice, #*p* < 0.05 MR409 treated mice vs. diabetic mice.

We used a high-resolution Doppler image system for small animal to determine heart valves and vascular calcification in aortic root. The ultrasound images of heart valve or vascular calcification manifests calcified spots or plaques (white) with abnormal increase in ultrasound density. Compared with WT control mice, high brightness calcified plaques with abnormal increased ultrasound density were seen in density of aortic valves (indicated with arrowhead) of diabetic mice, which disappeared in diabetic mice treated with MR409 (Figure 4). Furthermore, we used alizarin red and Von Kossa calcium staining to evaluate vascular calcification in the aortic root. Vascular calcium deposition was brown in Von Kossa staining and dark red in alizarin red staining. Compared with WT mice, diabetic mice had more brown staining areas in the aortic root section with Von Kossa staining (Figures 5A, C) and more dark red areas with alizarin red staining (*P* < 0.05; Figures 5B, D). MR409 treatment significantly reduced positive vascular staining area in both Von Kossa staining or alizarin red staining (*P* < 0.05; Figures 5B, D) in diabetic db/db mice. These results suggest that MR409 can inhibit vascular calcification in diabetes. ALP and Runx2 are two important molecules to regulate ectopic calcification (22, 23), ALP accelerates ectopic calcification by catalyzing hydrolysis of inorganic phosphate (23), Runx-2 is a major regulator of osteocyte differentiation to drive VC processes by the regulation of osteogenesis gene expression (22). We detected serum ALP activity and aortic expression of ALP and Runx2 by immunohistochemistry. Comparing with WT group, serum ALP activity significantly increased in diabetic mice, MR409 treatment restored ALP activity in diabetic mice (Figure 6A). Immunohistochemistry revealed that diabetic mice had more ALP and Runx2 expression, which were reduced in diabetic mice with MR409 treatment (Figures 6B–E).

### MR409 up-regulated Klotho expression in aorta of diabetic db/db mice

**FIGURE 7:**
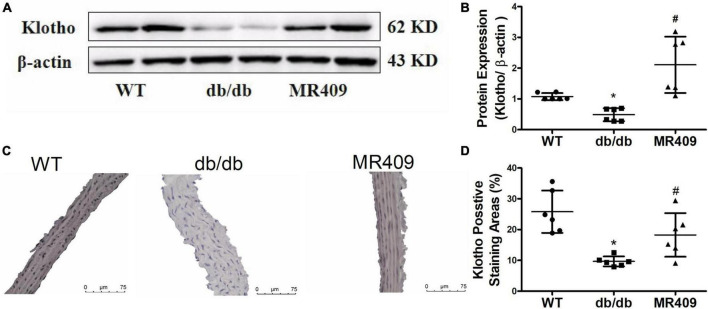
Treatment with MR409 upregulates the expression of Klotho in the aorta of diabetic db/de mice. The protein expression of Klotho was determined by Western blot **(A)**, the representative images of Klotho protein expression, determined by immunohistochemistry **(C)**, the quantitation of Klotho protein expression with Western blot **(B)** and immunohistochemistry **(D)**. *N* = 6, *p < 0.05 diabetic mice vs. WT mice, #*p* < 0.05 MR409 treated mice vs. diabetic mice.

Klotho is an anti-aging and anti-calcifying protein, which is reduced in chronic kidney diseases and diabetic mellitus (24, 25). Klotho expression significantly decreased in the aorta of db/db mice, as determined with Western blot and immunohistochemistry. MR409 treatment significantly increased aortic Klotho expression (Figure 7).

## Discussion

In the present study, we demonstrate that long-term treatment with GHRH-A MR409 significantly attenuated VC and vascular morphological injury, improved endothelial function but did not affect serum level of GH in db/db diabetic mice. The inhibitory effects of MR409 on VC was associated with a reduction in vascular ROS production and the expressions of osteogenic regulated gene Runx2 and ALP and an increase in anti-calcifying protein Klotho. These results confirm that GHRH-A may be a novel therapy for VC and VC associated vasculopathy.

Vascular calcification is a complex process that involves osteogenesis of vascular cells. VC represents a vascular aging phenotype which is commonly observed in the elderly people. Diabetes and chronic renal diseases are most common human disease associated with VC (2, 26), especially arterial medial calcification, because the patients suffer from a disturbed mineral and bone metabolism (27). Arterial medial calcification increases arterial stiffness, blood pressure and heart workload, impairs vascular compliance and vascular reactivity. Arterial medial calcification is an independent risk factor for development of stroke, cardiovascular diseases and mortality in type 2 diabetes mellitus (4, 28). Current anti-calcifying therapies may delay but can not effectively reduce existing arterial calcification and conduit vessel compliance (8). In addition, anti-calcifying therapies may cause detrimental side effects at the level of bone, as the molecular and cellular process for arterial calcification is similar to physiological mineralization (5, 8). We found that diabetic db/db mice at 20-week-old developed aortic medial calcification and aortic valve calcification, which were prevented by MR409 treatment.

MR409 is a synthetic peptide of 29 amino acids with the GHRH activity site of GHRH and adjacent peptide modifications that enhance its activity and stability (14). GHRH is a hypothalamic neuropeptide, the native GHRH stimulates the release of GH by binding to GHRH receptors in pituitary gland (29). It has been reported that in addition to pituitary cells, GHRH receptors are expressed in various extrapituitary cells (10). Consistent with previous findings (18), we found that long-term treatment with MR409 reduced VC but did not affect serum level of GH in diabetic db/db mice. These results suggest that anti-calcification and vasoprotective effects of MR409 may be achieved by directly binding to its receptors in vascular cells, independent of its effects on GH-regulation pathway. As GHRH agonists are not anti-calcifying agents, MR409 may avoid the side effects of activation of GH regulatory pathways and disturbed bone mineral metabolism. Thus GHRH-A may be a safe and novel agent for VC treatment.

The molecular and cellular mechanisms of VC are complex in diabetes (2, 30). Insulin resistance, hyperglycemia and hyperlipidemia are hallmarks of diabetes (2), which could be actively involved in pathological process of VC through the production of ROS and increased proinflammatory cytokines in vascular cells (1, 31). Accumulating evidence has shown that excessive production of ROS induces osteogenic transdifferentiation of VSMCs associated with increased expression of osteogenic markers, such as Runx2, ALP, and OCN (31, 32). Runx2 is an important transcription factor that regulates osteoblast differentiation (33, 34). It has been shown that ROS induces VSMC calcification through the upregulation of Runx2, and specific expression of Runx2 in VSMCs promotes osteogenic differentiation of VSMC and atherosclerotic calcification in the ApoE^–/–^ mice (32). ROS can activate redox sensitive transcription factor NF*k*B inflammatory pathway (35), which has been shown to increase expressions of osteogenic genes including Runx2 and ALP. Recently, Shen et al. (18) demonstrated that the activation of GHRH receptor signaling inhibits osteogenesis of VSMCs *in vitro* and VC *in vivo* by suppression of ROS-mediated NF*k*B/Runx2 pathway. In the present study, we found that ROS and osteogenic markers Runx2 and ALP increased in the aorta of diabetic db/db mice. MR409 treatment reduced ROS production and the expression of these osteogenic molecules, suggesting that MR409 may reduce VC *via* the inhibition of ROS/Runx2 in diabetes.

In the present study, MR409 did not affect blood level of glucose but significantly lowered serum levels of total triglyceride and total cholesterol in diabetic mice. The association of dyslipidemia and VC is well established, especially oxidized lipids or lipoprotein, such as oxidized phospholipids, which have shown to upregulate osteogenic-regulating gene expression and promote VC (36). Thus, lowering lipid profile may be another mechanism of MR409 inhibiting VC in diabetic mice.

It has been shown that anti-age protein Klotho has an important protective role against vascular dysfunction and VC (25). Klotho gene deficiency mice exhibit premature aging syndrome, accompanied with significant VC (24), and genetic overexpression of Klotho or administration of soluble Klotho rescues the Klotho-deficient phenotype and VC (37). Klotho is endowed with pleiotropic vasoprotective effects, such as the inhibition of oxidative stress and upregulation of eNOS expression in vascular cells. In addition, Klotho inhibits the osteogenic phenotype conversion of vascular progenitors or VSMCs through regulating Runx2 or mineral homeostasis (24). The patients with aging, diabetes and chronic renal diseases often have low plasma or tissue level of Klotho (38). In the present study, we found that expression of Klotho protein was downregulated in the aorta of diabetic mice, and MR409 treatment restored Klotho expression associated with reduction in vascular ROS, osteogenic protein expression of Runx2 and ALP. Thus, we surmise that attenuation of VC by MR409 may be through upregulation of Klotho, which inhibits ROS and Runx2-mediated osteogenic transdifferentiation of VSMCs in diabetes.

There are several limitations in our study. First, we used C57BL/6 mice as a wild type control group, although db/db mice are created on the background of C57BL/6 mice, C57BL/6 mice display stronger resistance to develop VC, heterozygote litters from some colony of db/db mice may be more suitable to use as the control mice. Next, although we have shown that treatment with MR409 can suppress VC and osteogenic-related genes expression in diabetic db/db mice, we have not yet determined a causal relationship between these osteogenic genes and MR409 attenuation of VC, which should be addressed in future study; Finally, we started to treat db/db mice at 12-week old, at this age, db/db mice may not develop significant VC. Thus, our results may only be applied for MR409 prevention of VC in diabetes, an intervention study that MR409 treatment was initiated after developed VC (for example, starting at 20-weeks old or late) may be required to address whether MR409 can be used to regress VC or clinically to treat VC patients.

In conclusion, the present study demonstrates for first time that treatment with GHRH-A MR409 effectively attenuates VC and heart valve calcification, accompanied by the improvement of endothelial function and vascular injury in diabetic db/db mice, an animal model that mimics type 2 diabetes in human. MR409 also increases protein expression of vascular Klotho and decreases ROS production and osteogenic genes Runx2 and ALP expressions without significant alteration in serum level of GH. These results suggest that GHRH-A MR409 could be a safe and novel approach to prevention and treatment of diabetes-induce VC and its associated vascular complications.

## Data availability statement

The original contributions presented in this study are included in this article/Supplementary material, further inquiries can be directed to the corresponding author.

## Ethics statement

The animal study was reviewed and approved by the Institutional Animal Care and Use Committee of Shenyang Medical College.

## Author contributions

H-LR contributed to the experimental design, data acquisition, and statistical analysis. RPC and RX contributed to data acquisition and statistical analysis. YZ and QX contributed to the pathological experiments. XZ contributed to the experimental design. RZC and WS contributed to the synthesis of MR409. AS contributed to the experimental design and reversed manuscript. M-SZ contributed to the conception and design the work, data interpretation, and drafted the manuscript. All authors contributed to the article and approved the submitted version.

## References

[1] Lee S Lee I Jeon J. Vascular calcification-new insights into its mechanism. *Int J Mol Sci.* (2020) 2020:21. 10.3390/ijms21082685 PMC7216228 32294899

[2] Stabley J Towler D. Arterial calcification in diabetes mellitus: preclinical models and translational implications. *Arterioscler Thromb Vasc Biol.* (2017) 37:205–17. 10.1161/ATVBAHA.116.306258 28062508 PMC5480317

[3] Lanzer P Hannan F Lanzer J Janzen J Raggi P Furniss D Medial arterial calcification: JACC state-of-the-art review. *J Am Coll Cardiol.* (2021) 78:1145–65. 34503684 10.1016/j.jacc.2021.06.049 PMC8439554

[4] Lanzer P Boehm M Sorribas V Thiriet M Janzen J Zeller T Medial vascular calcification revisited: review and perspectives. *Eur Heart J.* (2014) 35:1515–25. 10.1093/eurheartj/ehu163 24740885 PMC4072893

[5] Demer L Tintut Y. Vascular calcification: pathobiology of a multifaceted disease. *Circulation.* (2008) 117:2938–48. 18519861 10.1161/CIRCULATIONAHA.107.743161 PMC4431628

[6] Vieceli Dalla Sega F Fortini F Severi P Rizzo P Gardi I Cimaglia P Cardiac calcifications: phenotypes, mechanisms, clinical and prognostic implications. *Biology.* (2022) 2022:11. 10.3390/biology11030414 35336788 PMC8945469

[7] Avogaro A Fadini G. Mechanisms of ectopic calcification: implications for diabetic vasculopathy. *Cardiovasc Diagn Ther.* (2015) 5:343–52. 26543821 10.3978/j.issn.2223-3652.2015.06.05 PMC4609906

[8] Van den Branden A Verhulst A D’Haese P Opdebeeck B. New therapeutics targeting arterial media calcification: friend or foe for bone mineralization? *Metabolites.* (2022) 2022:12. 10.3390/metabo12040327 PMC9027727 35448514

[9] Fridlyand L Tamarina N Schally A Philipson L. Growth hormone-releasing hormone in diabetes. *Front Endocrinol.* (2016) 7:129. 10.3389/fendo.2016.00129 27777568 PMC5056186

[10] Zhang X Cui T He J Wang H Cai R Popovics P Beneficial effects of growth hormone-releasing hormone agonists on rat INS-1 cells and on streptozotocin-induced NOD/SCID mice. *Proc Natl Acad Sci USA.* (2015) 112:13651–6. 10.1073/pnas.1518540112 26474831 PMC4640729

[11] Thounaojam M Powell F Patel S Gutsaeva D Tawfik A Smith S Protective effects of agonists of growth hormone-releasing hormone (GHRH) in early experimental diabetic retinopathy. *Proc Natl Acad Sci USA.* (2017) 114:13248–53. 10.1073/pnas.1718592114 29180438 PMC5740669

[12] Liu Y Yang J Che X Huang J Zhang X Fu X Agonistic analog of growth hormone-releasing hormone promotes neurofunctional recovery and neural regeneration in ischemic stroke. *Proc Natl Acad Sci USA.* (2021) 2021:118. 10.1073/pnas.2109600118 34782465 PMC8617525

[13] Xiang P Jing W Lin Y Liu Q Shen J Hu X Improvement of cardiac and systemic function in old mice by agonist of growth hormone-releasing hormone. *J Cell Physiol.* (2021) 236:8197–207. 10.1002/jcp.30490 34224586

[14] Cai R Schally A Cui T Szalontay L Halmos G Sha W Synthesis of new potent agonistic analogs of growth hormone-releasing hormone (GHRH) and evaluation of their endocrine and cardiac activities. *Peptides.* (2014) 52:104–12. 10.1016/j.peptides.2013.12.010 24373935 PMC4745889

[15] Schally A Zhang X Cai R Hare J Granata R Bartoli M. Actions and potential therapeutic applications of growth hormone-releasing hormone agonists. *Endocrinology.* (2019) 160:1600–12. 31070727 10.1210/en.2019-00111

[16] Kanashiro-Takeuchi R Takeuchi L Rick F Dulce R Treuer A Florea V Activation of growth hormone releasing hormone (GHRH) receptor stimulates cardiac reverse remodeling after myocardial infarction (MI). *Proc Natl Acad Sci USA.* (2012) 109:559–63. 10.1073/pnas.1119203109 22203988 PMC3258609

[17] Dulce R Kanashiro-Takeuchi R Takeuchi L Salerno A Wanschel A Kulandavelu S Synthetic growth hormone-releasing hormone agonist ameliorates the myocardial pathophysiology characteristic of HFpEF. *Cardiovasc Res.* (2022). [Epub ahead of print]. 10.1093/cvr/cvac098 35704032 PMC10202441

[18] Shen J Zhang N Lin Y Xiang P Liu X Shan P Regulation of vascular calcification by growth hormone-releasing hormone and its agonists. *Circ Res.* (2018) 122:1395–408. 29618597 10.1161/CIRCRESAHA.117.312418 PMC5948169

[19] Woodward H Zhu D Hadoke P MacRae V. Regulatory role of sex hormones in cardiovascular calcification. *Int J Mol Sci.* (2021) 2021:22. 10.3390/ijms22094620 PMC8125640 33924852

[20] Bostrom K Jumabay M Matveyenko A Nicholas S Yao Y. Activation of vascular bone morphogenetic protein signaling in diabetes mellitus. *Circ Res.* (2011) 108:446–57. 21193740 10.1161/CIRCRESAHA.110.236596 PMC3042480

[21] Yang X Wang H Huang Y Huang J Ren H Xu Q Myeloid angiotensin II type 1 receptor mediates macrophage polarization and promotes vascular injury in DOCA/salt hypertensive mice. *Front Pharmacol.* (2022) 13:879693. 10.3389/fphar.2022.879693 35721173 PMC9204513

[22] Lin M Chen T Wallingford M Nguyen N Yamada S Sawangmake C Runx2 deletion in smooth muscle cells inhibits vascular osteochondrogenesis and calcification but not atherosclerotic lesion formation. *Cardiovasc Res.* (2016) 112:606–16. 10.1093/cvr/cvw205 27671804 PMC5079276

[23] Haarhaus M Cianciolo G Barbuto S La Manna G Gasperoni L Tripepi G Alkaline phosphatase: an old friend as treatment target for cardiovascular and mineral bone disorders in chronic kidney disease. *Nutrients.* (2022) 2022:14. 10.3390/nu14102124 35631265 PMC9144546

[24] Hu M Shi M Zhang J Quinones H Griffith C Kuro-o M Klotho deficiency causes vascular calcification in chronic kidney disease. *J Am Soc Nephrol.* (2011) 22:124–36. 21115613 10.1681/ASN.2009121311 PMC3014041

[25] Prud’homme G Kurt M Wang Q. Pathobiology of the Klotho antiaging protein and therapeutic considerations. *Front Aging.* (2022) 3:931331. 10.3389/fragi.2022.931331 35903083 PMC9314780

[26] Yamada S Giachelli C. Vascular calcification in CKD-MBD: roles for phosphate, FGF23, and klotho. *Bone.* (2017) 100:87–93. 27847254 10.1016/j.bone.2016.11.012 PMC5429216

[27] Chen Y Zhao X Wu H. Arterial stiffness: a focus on vascular calcification and its link to bone mineralization. *Arterioscler Thromb Vasc Biol.* (2020) 40:1078–93. 10.1161/ATVBAHA.120.313131 32237904 PMC7199843

[28] Ogunmoroti O Osibogun O Mathews L Esuruoso O Ndumele C Okunrintemi V Favorable cardiovascular health is associated with lower prevalence, incidence, extent, and progression of extracoronary calcification: MESA. *Circ Cardiovasc Imaging.* (2022) 15:e013762. 10.1161/CIRCIMAGING.121.013762 35290079 PMC9179934

[29] Devesa J. The complex world of regulation of pituitary growth hormone secretion: the role of ghrelin, klotho, and nesfatins in it. *Front Endocrinol.* (2021) 12:636403. 10.3389/fendo.2021.636403 33776931 PMC7991839

[30] Ghosh S Luo D He W Chen J Su X Huang H. Diabetes and calcification: the potential role of anti-diabetic drugs on vascular calcification regression. *Pharmacol Res.* (2020) 158:104861. 10.1016/j.phrs.2020.104861 32407954

[31] Toth A Balogh E Jeney V. Regulation of vascular calcification by reactive oxygen species. *Antioxidants.* (2020) 2020:9. 10.3390/antiox9100963 PMC7599480 33049989

[32] Furmanik M Chatrou M van Gorp R Akbulut A Willems B Schmidt H Reactive oxygen-forming nox5 links vascular smooth muscle cell phenotypic switching and extracellular vesicle-mediated vascular calcification. *Circ Res.* (2020) 127:911–27. 10.1161/CIRCRESAHA.119.316159 32564697

[33] Li P Wang Y Liu X Liu B Wang Z Xie F Loss of PARP-1 attenuates diabetic arteriosclerotic calcification via stat1/runx2 axis. *Cell Death Dis.* (2020) 11:22. 10.1038/s41419-019-2215-8 PMC6954221 31924749

[34] Li W Feng W Su X Luo D Li Z Zhou Y SIRT6 protects vascular smooth muscle cells from osteogenic transdifferentiation via runx2 in chronic kidney disease. *J Clin Invest.* (2022) 2022:132. 10.1172/JCI150051 34793336 PMC8718147

[35] He X Wang Z Wei L Cheng X Chen L Gao F Indoxyl sulfate promotes osteogenic differentiation of vascular smooth muscle cells by miR-155-5p-dependent downregulation of matrix Gla protein via ROS/NF-kappaB signaling. *Exp Cell Res.* (2020) 397:112301. 10.1016/j.yexcr.2020.112301 32979364

[36] Tintut Y Hsu J Demer L. Lipoproteins in cardiovascular calcification: potential targets and challenges. *Front Cardiovasc Med.* (2018) 5:172. 10.3389/fcvm.2018.00172 30533416 PMC6265366

[37] Yu L Li M. Roles of klotho and stem cells in mediating vascular calcification (review). *Exp Ther Med.* (2020) 20:124. 10.3892/etm.2020.9252 PMC7523279 33005250

[38] Zubkiewicz-Kucharska A Wikiera B Noczynska A. Soluble klotho is decreased in children with type 1 diabetes and correlated with metabolic control. *Front Endocrinol.* (2021) 12:709564. 10.3389/fendo.2021.7095 PMC8484787 34603200

