# Supplementary material for: Growth hormone-releasing hormone agonist attenuates vascular calcification in diabetic db/db mice
Source: Front Cardiovasc Med. 2023 Jan 18;10:1102525. doi: 10.3389/fcvm.2023.1102525 (PMC9889365; doi:10.3389/fcvm.2023.1102525)
Supplement: Supplementary file 1 [file Data_Sheet_1.pdf]

Figure 1

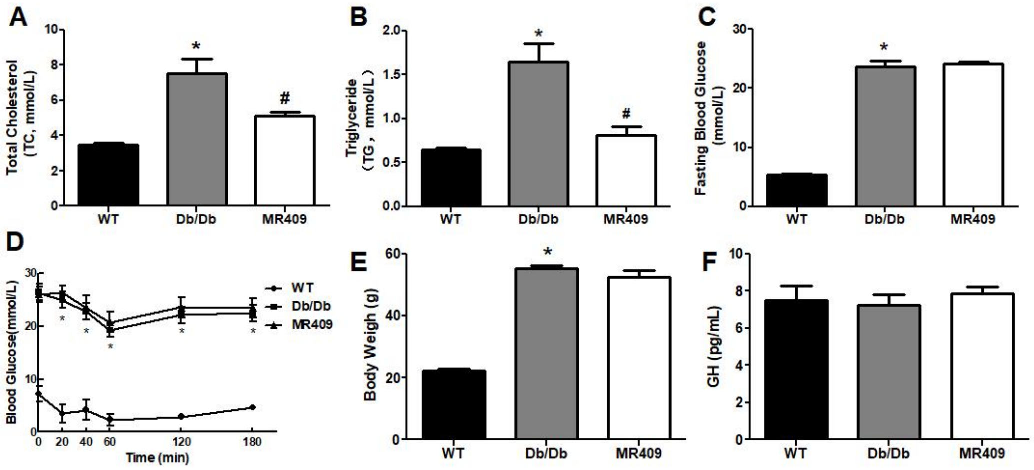

Fig 1A

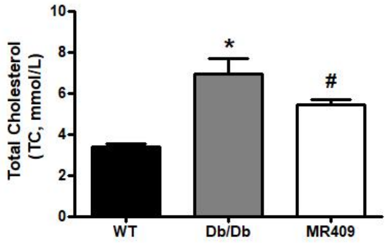

A	B	C
WT	Db/Db	MR409
Y	Y	Y
3.096751	7.269492	5.963535
3.621624	6.062284	5.248731
3.414605	5.867349	5.097860
3.528148	10.917120	4.616930
3.753727	8.849116	4.857395
3.395823	6.272233	4.977628

1	Table Analyzed	Data 1
2	Column A	WT
3	vs	vs
4	Column B	Db/Db
5		
6	Unpaired t test	
7	P value	0.0003
8	P value summary	***
9	Are means signif. different? (P < 0.05)	Yes
10	One- or two-tailed P value?	One-tailed
11	t, df	t=4.982 df=10
12		
13	How big is the difference?	
14	Mean ± SEM of column A	3.468 ?0.09215 N=6
15	Mean ± SEM of column B	7.540 ?0.8120 N=6
16	Difference between means	-4.071 ?0.8172
17	95% confidence interval	-5.892 to -2.250
18	R squared	0.7128
19		
20	F test to compare variances	
21	F,DFn, Dfd	77.65, 5, 5
22	P value	0.0002
23	P value summary	***
24	Are variances significantly different?	Yes

1	Table Analyzed	Data 1
2	Column B	Db/Db
3	vs	vs
4	Column C	MR409
5		
6	Unpaired t test	
7	P value	0.0080
8	P value summary	**
9	Are means signif. different? (P < 0.05)	Yes
10	One- or two-tailed P value?	One-tailed
11	t, df	t=2.894 df=10
12		
13	How big is the difference?	
14	Mean ± SEM of column B	7.540 ?0.8120 N=6
15	Mean ± SEM of column C	5.127 ?0.1889 N=6
16	Difference between means	2.413 ?0.8337
17	95% confidence interval	0.5551 to 4.270
18	R squared	0.4558
19		
20	F test to compare variances	
21	F,DFn, Dfd	18.48, 5, 5
22	P value	0.0061
23	P value summary	**
24	Are variances significantly different?	Yes

Fig 1B

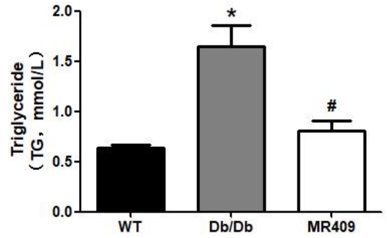

A	B	C
WT	Db/Db	MR409
Y	Y	Y
3.096751	7.269492	5.963535
3.621624	6.062284	5.248731
3.414605	5.867349	5.097860
3.528148	10.917120	4.616930
3.753727	8.849116	4.857395
3.395823	6.272233	4.977628

1	Table Analyzed	Data 1
2	Column A	WT
3	vs	vs
4	Column B	Db/Db
5		
6	Unpaired t test	
7	P value	0.0003
8	P value summary	***
9	Are means signif. different? (P < 0.05)	Yes
10	One- or two-tailed P value?	One-tailed
11	t, df	t=4.886 df=10
12		
13	How big is the difference?	
14	Mean ± SEM of column A	0.6410 ?0.02819 N=6
15	Mean ± SEM of column B	1.649 ?0.2043 N=6
16	Difference between means	-1.008 ?0.2062
17	95% confidence interval	-1.467 to -0.5481
18	R squared	0.7048
19		
20	F test to compare variances	
21	F,DFn, Dfd	52.49, 5, 5
22	P value	0.0005
23	P value summary	***
24	Are variances significantly different?	Yes

1	Table Analyzed	Data 1
2	Column B	Db/Db
3	vs	vs
4	Column C	MR409
5		
6	Unpaired t test	
7	P value	0.0020
8	P value summary	**
9	Are means signif. different? (P < 0.05)	Yes
10	One- or two-tailed P value?	One-tailed
11	t, df	t=3.722 df=10
12		
13	How big is the difference?	
14	Mean ± SEM of column B	1.649 ?0.2043 N=6
15	Mean ± SEM of column C	0.8102 ?0.09498 N=6
16	Difference between means	0.8384 ?0.2253
17	95% confidence interval	0.3365 to 1.340
18	R squared	0.5807
19		
20	F test to compare variances	
21	F,DFn, Dfd	4.625, 5, 5
22	P value	0.1182
23	P value summary	ns
24	Are variances significantly different?	No

Fig 1C

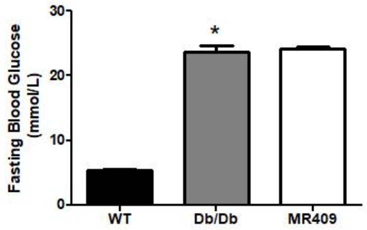

	A	B	C
	WT	Db/Db	MR409
	Y	Y	Y
1	5.3	23.3	24.1
2	5.5	21.3	22.9
3	5.4	27.6	24.5
4	5.6	25.7	23.6
5	5.3	22.9	25.3
6	5.4	21.2	24.4

1	Table Analyzed	Data 1
2	Column A	WT
3	vs	vs
4	Column B	Db/Db
5		
6	Unpaired t test	
7	P value	< 0.0001
8	P value summary	***
9	Are means signif. different? (P < 0.05)	Yes
10	One- or two-tailed P value?	One-tailed
11	t, df	t=17.65 df=10
12		
13	How big is the difference?	
14	Mean ± SEM of column A	5.417 ?0.04773 N=6
15	Mean ± SEM of column B	23.67 ?1.033 N=6
16	Difference between means	-18.25 ?1.034
17	95% confidence interval	-20.55 to -15.95
18	R squared	0.9689
19		
20	F test to compare variances	
21	F,DFn, Dfd	468.5, 5, 5
22	P value	< 0.0001
23	P value summary	***
24	Are variances significantly different?	Yes

1	Table Analyzed	Data 1
2	Column B	Db/Db
3	vs	vs
4	Column C	MR409
5		
6	Unpaired t test	
7	P value	0.3383
8	P value summary	ns
9	Are means signif. different? (P < 0.05)	No
10	One- or two-tailed P value?	One-tailed
11	t, df	t=0.4297 df=10
12		
13	How big is the difference?	
14	Mean ± SEM of column B	23.67 ?1.033 N=6
15	Mean ± SEM of column C	24.13 ?0.3353 N=6
16	Difference between means	-0.4667 ?1.086
17	95% confidence interval	-2.886 to 1.953
18	R squared	0.01813
19		
20	F test to compare variances	
21	F,DFn, Dfd	9.490, 5, 5
22	P value	0.0274
23	P value summary	*
24	Are variances significantly different?	Yes

Fig 1D

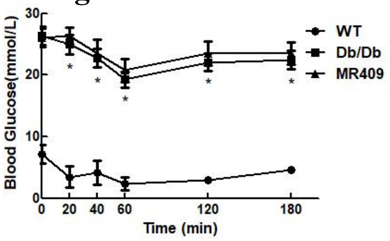

Table format: XY	X	A		B		C	
	Time	WT		Db/Db		MR409	
	X	Mean	SEM	Mean	SEM	Mean	SEM
1 Title	0	7.175	1.480076	26.409090	1.510000	26.016670	1.500497
2 Title	20	3.450	1.697793	24.927270	1.510000	26.325000	1.363492
3 Title	40	4.130	1.901480	22.750000	1.530000	23.490000	2.210048
4 Title	60	2.330	1.075000	19.260000	1.250000	20.760000	1.910278
5 Title	120	2.880	0.292617	22.060000	1.470000	23.530000	1.960060
6 Title	180	4.650	0.499166	22.430000	1.530000	23.480000	1.807439

1	Table Analyzed	Data 1
2	Column A	WT
3	vs	vs
4	Column B	Db/Db
5		
6	Unpaired t test	
7	P value	< 0.0001
8	P value summary	***
9	Are means signif. different? (P < 0.05)	Yes
10	One- or two-tailed P value?	One-tailed
11	t, df	t=15.34 df=10
12		
13	How big is the difference?	
14	Mean ± SEM of column A	4.103 ?0.7024 N=6
15	Mean ± SEM of column B	22.97 ?1.010 N=6
16	Difference between means	-18.87 ?1.230
17	95% confidence interval	-21.61 to -16.13
18	R squared	0.9592
19		
20	F test to compare variances	
21	F,DFn, Dfd	2.067, 5, 5
22	P value	0.4446
23	P value summary	ns
24	Are variances significantly different?	No

1	Table Analyzed	Data 1
2	Column B	Db/Db
3	vs	vs
4	Column C	MR409
5		
6	Unpaired t test	
7	P value	0.2396
8	P value summary	ns
9	Are means signif. different? (P < 0.05)	No
10	One- or two-tailed P value?	One-tailed
11	t, df	t=0.7349 df=10
12		
13	How big is the difference?	
14	Mean ± SEM of column B	22.97 ?1.010 N=6
15	Mean ± SEM of column C	23.93 ?0.8306 N=6
16	Difference between means	-0.9609 ?1.307
17	95% confidence interval	-3.874 to 1.952
18	R squared	0.05124
19		
20	F test to compare variances	
21	F,DFn, Dfd	1.478, 5, 5
22	P value	0.6785
23	P value summary	ns
24	Are variances significantly different?	No

Fig 1E

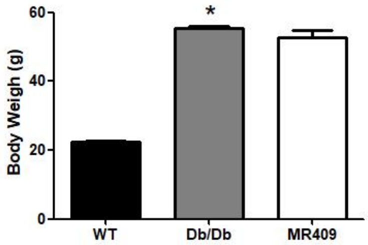

	A	B	C
	WT	Db/Db	MR409
	Y	Y	Y
1	21.38	56.20	58.00
2	21.77	54.20	47.90
3	21.54	53.90	45.30
4	21.84	53.70	51.70
5	22.18	58.30	54.10
6	24.90	55.90	58.40

1	Table Analyzed	Data 1
2	Column A	WT
3	vs	vs
4	Column B	Db/Db
5		
6	Unpaired t test	
7	P value	< 0.0001
8	P value summary	***
9	Are means signif. different? (P < 0.05)	Yes
10	One- or two-tailed P value?	One-tailed
11	t, df	t=36.58 df=10
12		
13	How big is the difference?	
14	Mean ± SEM of column A	22.27 ?0.5380 N=6
15	Mean ± SEM of column B	55.37 ?0.7274 N=6
16	Difference between means	-33.10 ?0.9048
17	95% confidence interval	-35.11 to -31.08
18	R squared	0.9926
19		
20	F test to compare variances	
21	F,DFn, Dfd	1.828, 5, 5
22	P value	0.5241
23	P value summary	ns
24	Are variances significantly different?	No

1	Table Analyzed	Data 1
2	Column B	Db/Db
3	vs	vs
4	Column C	MR409
5		
6	Unpaired t test	
7	P value	0.1246
8	P value summary	ns
9	Are means signif. different? (P < 0.05)	No
10	One- or two-tailed P value?	One-tailed
11	t, df	t=1.224 df=10
12		
13	How big is the difference?	
14	Mean ± SEM of column B	55.37 ?0.7274 N=6
15	Mean ± SEM of column C	52.57 ?2.170 N=6
16	Difference between means	2.800 ?2.288
17	95% confidence interval	-2.299 to 7.899
18	R squared	0.1302
19		
20	F test to compare variances	
21	F,DFn, Dfd	8.898, 5, 5
22	P value	0.0315
23	P value summary	*
24	Are variances significantly different?	Yes

Fig 1F

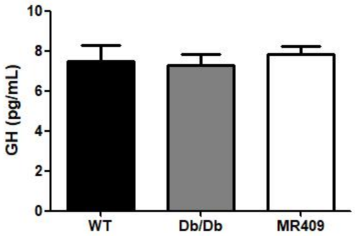

	A	B	C
	WT	Db/Db	MR409
	Y	Y	Y
1	4.3384	7.7801	7.2406
2	6.9888	9.2573	7.6042
3	6.9221	6.9246	8.9130
4	9.2369	5.8808	6.7293
5	9.9040	8.0103	9.0936
6	7.5841	5.7440	7.4960

1	Table Analyzed	Data 1
2	Column A	WT
3	vs	vs
4	Column B	Db/Db
5		
6	Unpaired t test	
7	P value	0.4093
8	P value summary	ns
9	Are means signif. different? (P < 0.05)	No
10	One- or two-tailed P value?	One-tailed
11	t, df	t=0.2354 df=10
12		
13	How big is the difference?	
14	Mean ± SEM of column A	7.496 ?0.8040 N=6
15	Mean ± SEM of column B	7.266 ?0.5518 N=6
16	Difference between means	0.2295 ?0.9752
17	95% confidence interval	-1.943 to 2.402
18	R squared	0.005510
19		
20	F test to compare variances	
21	F,DFn, Dfd	2.123, 5, 5
22	P value	0.4282
23	P value summary	ns
24	Are variances significantly different?	No

1	Table Analyzed	Data 1
2	Column B	Db/Db
3	vs	vs
4	Column C	MR409
5		
6	Unpaired t test	
7	P value	0.2048
8	P value summary	ns
9	Are means signif. different? (P < 0.05)	No
10	One- or two-tailed P value?	One-tailed
11	t, df	t=0.8605 df=10
12		
13	How big is the difference?	
14	Mean ± SEM of column B	7.266 ?0.5518 N=6
15	Mean ± SEM of column C	7.846 ?0.3868 N=6
16	Difference between means	-0.5799 ?0.6739
17	95% confidence interval	-2.081 to 0.9216
18	R squared	0.06895
19		
20	F test to compare variances	
21	F,DFn, Dfd	2.035, 5, 5
22	P value	0.4542
23	P value summary	ns
24	Are variances significantly different?	No

Figure 2

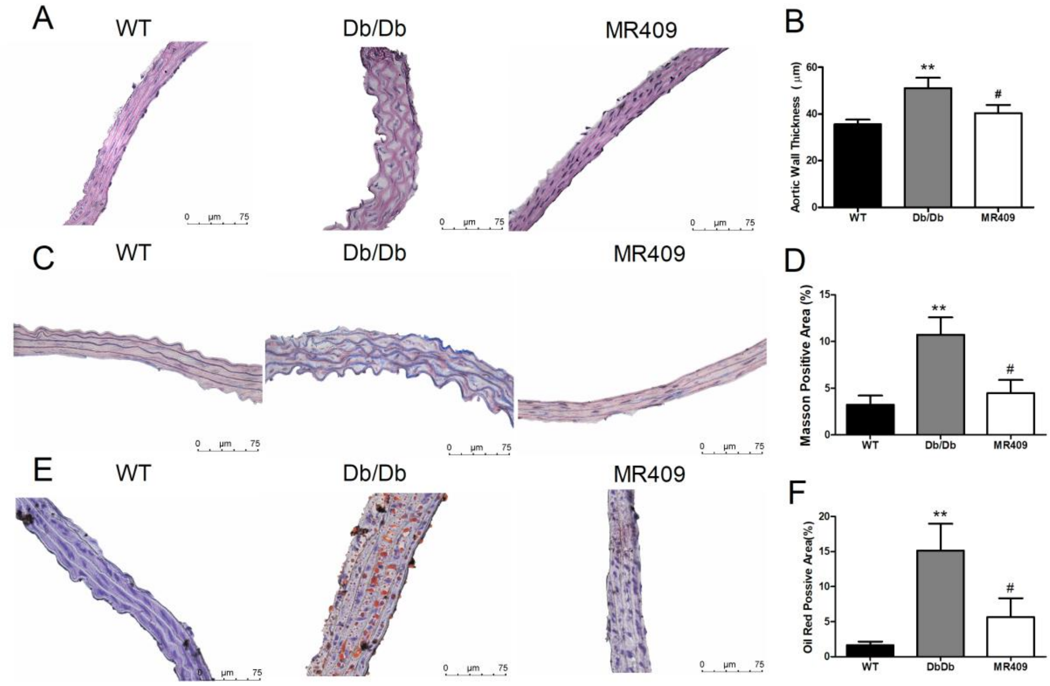

Fig 2A-WT

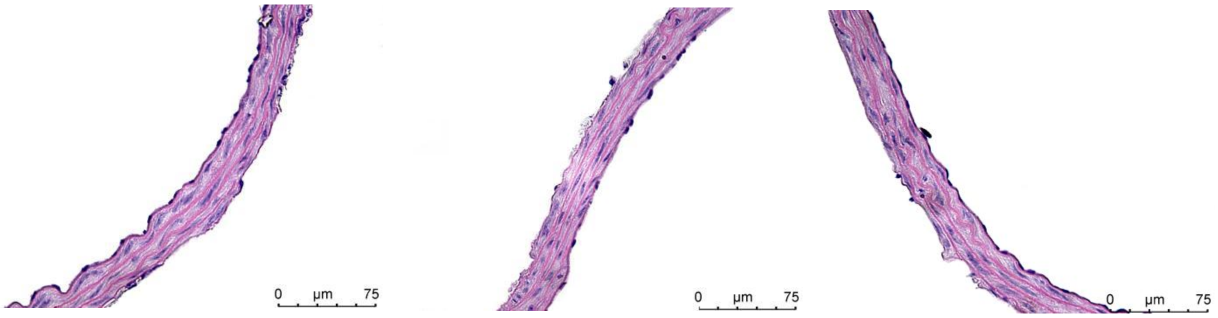

Fig 2A-Db/Db

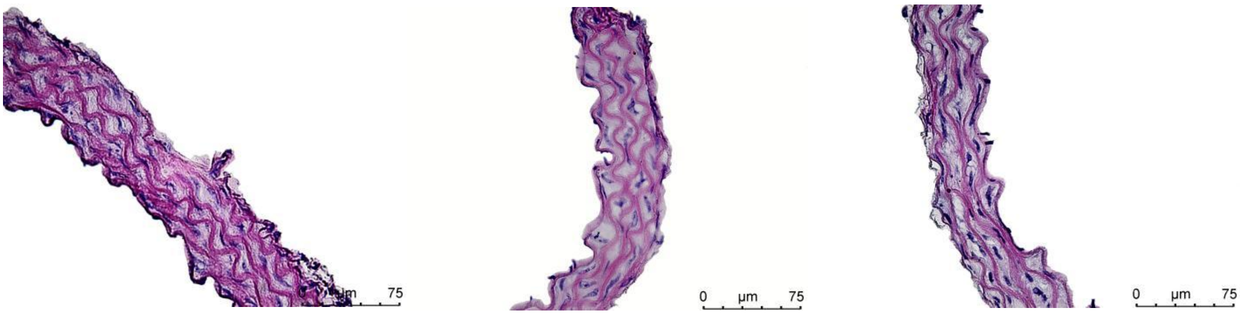

Fig 2A-MR409

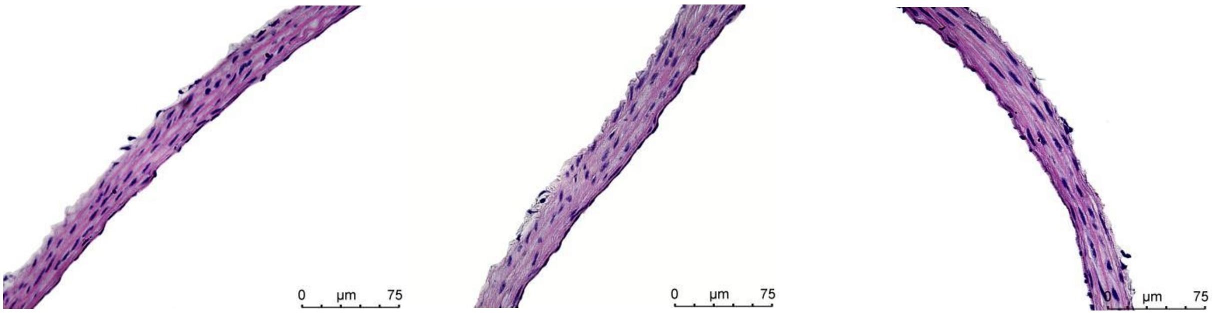

Fig 2B

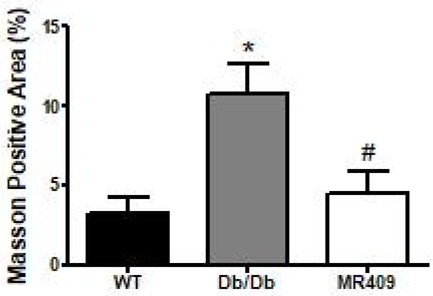

	A	B	C
	WT	Db/Db	MR409
	Y	Y	Y
1	42.339	53.863	40.047
2	37.566	67.045	36.569
3	41.198	55.845	41.338
4	34.332	77.842	47.144
5	31.250	51.725	38.327
6	31.106	50.900	40.393

1	Table Analyzed	Data 1
2	Column A	WT
3	vs	vs
4	Column B	Db/Db
5		
6	Unpaired t test	
7	P value	0.0003
8	P value summary	***
9	Are means signif. different? (P < 0.05)	Yes
10	One- or two-tailed P value?	One-tailed
11	t, df	t=4.841 df=10
12		
13	How big is the difference?	
14	Mean ± SEM of column A	36.30 ?1.987 N=6
15	Mean ± SEM of column B	59.54 ?4.370 N=6
16	Difference between means	-23.24 ?4.800
17	95% confidence interval	-33.93 to -12.54
18	R squared	0.7009
19		
20	F test to compare variances	
21	F,DFn, Dfd	4.838, 5, 5
22	P value	0.1086
23	P value summary	ns
24	Are variances significantly different?	No

1	Table Analyzed	Data 1
2	Column B	Db/Db
3	vs	vs
4	Column C	MR409
5		
6	Unpaired t test	
7	P value	0.0011
8	P value summary	**
9	Are means signif. different? (P < 0.05)	Yes
10	One- or two-tailed P value?	One-tailed
11	t, df	t=4.098 df=10
12		
13	How big is the difference?	
14	Mean ± SEM of column B	59.54 ?4.370 N=6
15	Mean ± SEM of column C	40.64 ?1.473 N=6
16	Difference between means	18.90 ?4.612
17	95% confidence interval	8.626 to 29.18
18	R squared	0.6268
19		
20	F test to compare variances	
21	F,DFn, Dfd	8.797, 5, 5
22	P value	0.0323
23	P value summary	*
24	Are variances significantly different?	Yes

Fig 2C

WT Db/Db MR409

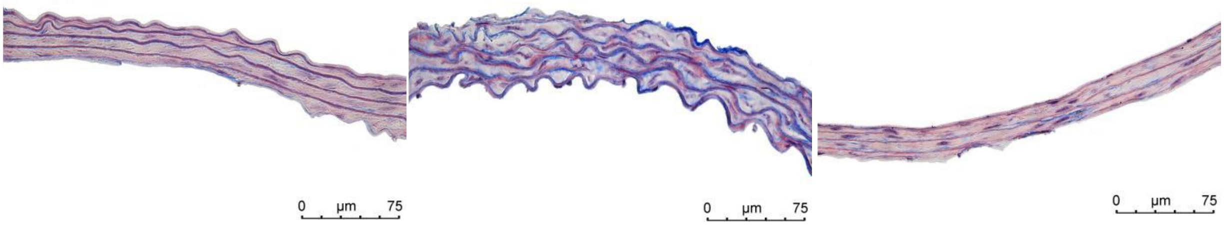

Fig 2D

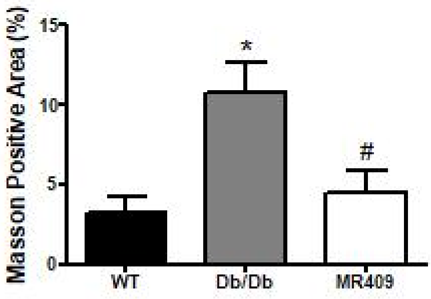

	A	B	C
	WT	Db/Db	MR409
	Y	Y	Y
1	2.94	15.27	7.27
2	6.13	8.12	1.89
3	1.64	8.08	1.19
4	6.37	4.25	2.32
5	0.59	15.26	9.74
6	1.56	13.43	4.58

1	Table Analyzed	Data 1
2	Column A	WT
3	vs	vs
4	Column B	Db/Db
5		
6	Unpaired t test	
7	P value	0.0026
8	P value summary	**
9	Are means signif. different? (P < 0.05)	Yes
10	One- or two-tailed P value?	One-tailed
11	t, df	t=3.551 df=10
12		
13	How big is the difference?	
14	Mean ± SEM of column A	3.205 ?1.011 N=6
15	Mean ± SEM of column B	10.74 ?1.864 N=6
16	Difference between means	-7.530 ?2.120
17	95% confidence interval	-12.25 to -2.806
18	R squared	0.5577
19		
20	F test to compare variances	
21	F,DFn, Dfd	3.403, 5, 5
22	P value	0.2051
23	P value summary	ns
24	Are variances significantly different?	No

1	Table Analyzed	Data 1
2	Column B	Db/Db
3	vs	vs
4	Column C	MR409
5		
6	Unpaired t test	
7	P value	0.0115
8	P value summary	*
9	Are means signif. different? (P < 0.05)	Yes
10	One- or two-tailed P value?	One-tailed
11	t, df	t=2.685 df=10
12		
13	How big is the difference?	
14	Mean ± SEM of column B	10.74 ?1.864 N=6
15	Mean ± SEM of column C	4.498 ?1.386 N=6
16	Difference between means	6.237 ?2.323
17	95% confidence interval	1.061 to 11.41
18	R squared	0.4188
19		
20	F test to compare variances	
21	F,DFn, Dfd	1.808, 5, 5
22	P value	0.5315
23	P value summary	ns
24	Are variances significantly different?	No

Fig 2E

WT Db/Db MR409

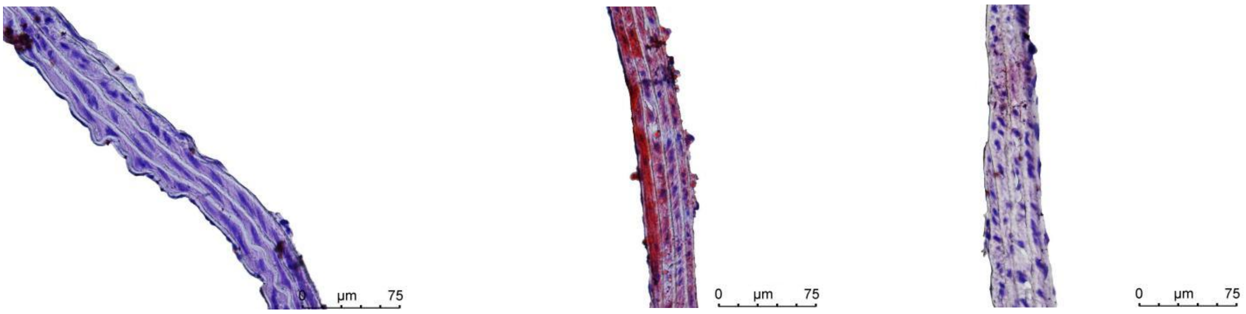

Fig 2F

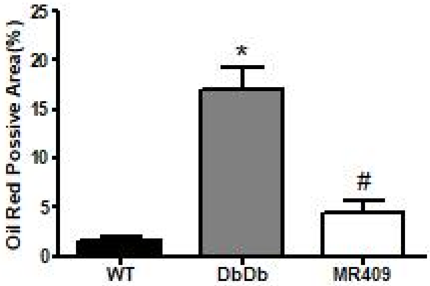

	A	B	C
	WT	DbDb	MR409
	Y	Y	Y
1	1.16	11.89	6.20
2	2.28	27.45	1.79
3	1.33	14.35	5.85
4	0.04	19.03	3.19
5	3.19	12.95	1.25
6	1.76	15.87	8.79

	Table Analyzed	Data 1
1	Table Analyzed	Data 1
2	Column A	WT
3	vs	vs
4	Column B	DbDb
5		
6	Unpaired t test	
7	P value	< 0.0001
8	P value summary	***
9	Are means signif. different? (P < 0.05)	Yes
10	One- or two-tailed P value?	One-tailed
11	t, df	t=6.429 df=10
12		
13	How big is the difference?	
14	Mean ± SEM of column A	1.627 ?0.4363 N=6
15	Mean ± SEM of column B	16.92 ?2.339 N=6
16	Difference between means	-15.30 ?2.379
17	95% confidence interval	-20.60 to -9.996
18	R squared	0.8052
19		
20	F test to compare variances	
21	F,DFn, Dfd	28.73, 5, 5
22	P value	0.0022
23	P value summary	**
24	Are variances significantly different?	Yes

	Table Analyzed	Data 1
1	Table Analyzed	Data 1
2	Column B	DbDb
3	vs	vs
4	Column C	MR409
5		
6	Unpaired t test	
7	P value	0.0004
8	P value summary	***
9	Are means signif. different? (P < 0.05)	Yes
10	One- or two-tailed P value?	One-tailed
11	t, df	t=4.727 df=10
12		
13	How big is the difference?	
14	Mean ± SEM of column B	16.92 ?2.339 N=6
15	Mean ± SEM of column C	4.512 ?1.194 N=6
16	Difference between means	12.41 ?2.626
17	95% confidence interval	6.561 to 18.26
18	R squared	0.6908
19		
20	F test to compare variances	
21	F,DFn, Dfd	3.840, 5, 5
22	P value	0.1661
23	P value summary	ns
24	Are variances significantly different?	No

Figure 3

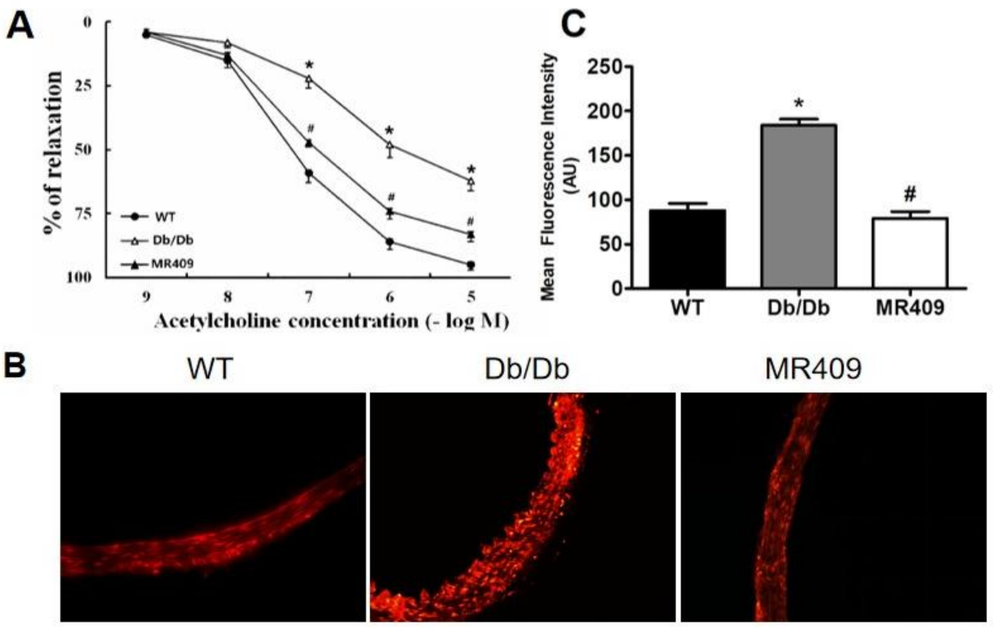

3A

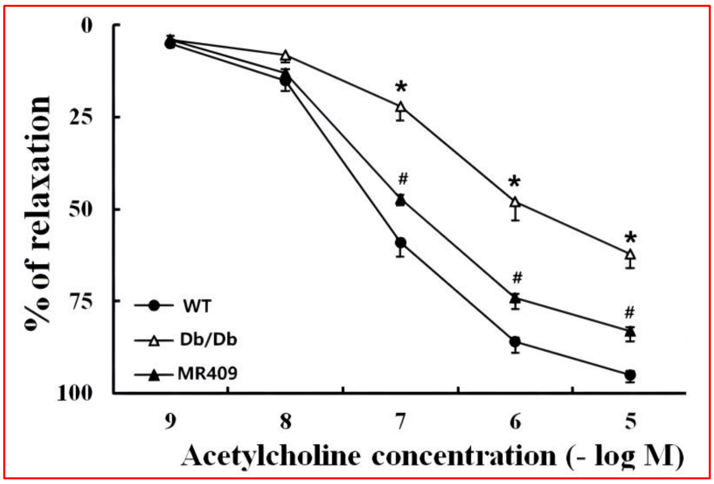

此处原始数据暂无

Fig 3B

WT	Db/Db	MR409

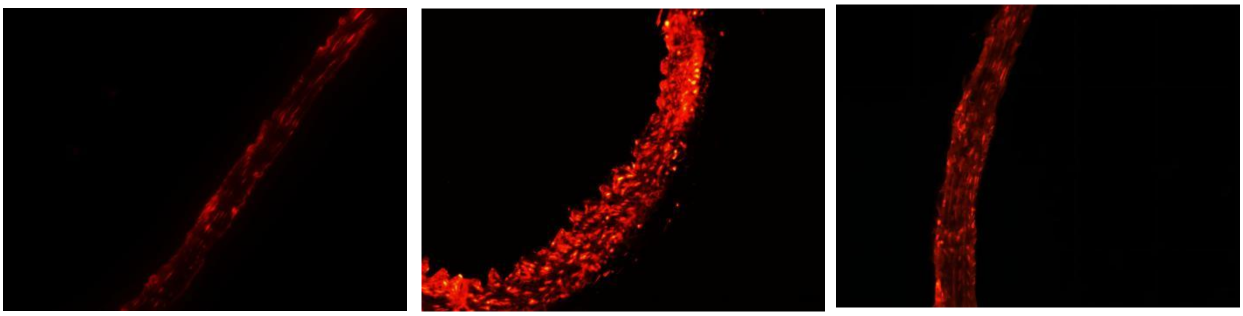

Fig 3C

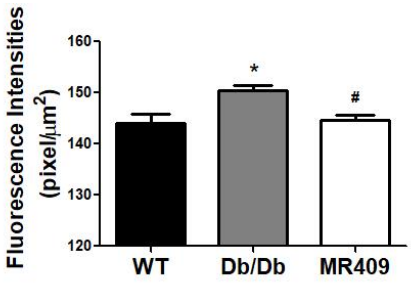

	A	B	C
	WT	Db/Db	MR409
	Y	Y	Y
1	132.770000	148.434800	141.337400
2	145.345300	151.698900	144.440100
3	144.835100	149.102500	147.855000
4	143.906200	150.335200	148.389700
5	146.945400	155.334500	141.224400
6	144.151300	152.848300	144.611800

1	Table Analyzed	Data 1
2	Column A	WT
3	vs	vs
4	Column B	Db/Db
5		
6	Unpaired t test	
7	P value	0.0026
8	P value summary	**
9	Are means signif. different? (P < 0.05)	Yes
10	One- or two-tailed P value?	One-tailed
11	t, df	t=3.550 df=10
12		
13	How big is the difference?	
14	Mean ± SEM of column A	143.0 ?2.092 N=6
15	Mean ± SEM of column B	151.3 ?1.046 N=6
16	Difference between means	-8.300 ?2.338
17	95% confidence interval	-13.51 to -3.090
18	R squared	0.5575
19		
20	F test to compare variances	
21	F,DFn, Dfd	4.001, 5, 5
22	P value	0.1543
23	P value summary	ns
24	Are variances significantly different?	No

1	Table Analyzed	Data 1
2	Column B	Db/Db
3	vs	vs
4	Column C	MR409
5		
6	Unpaired t test	
7	P value	0.0011
8	P value summary	**
9	Are means signif. different? (P < 0.05)	Yes
10	One- or two-tailed P value?	One-tailed
11	t, df	t=4.077 df=10
12		
13	How big is the difference?	
14	Mean ± SEM of column B	151.3 ?1.046 N=6
15	Mean ± SEM of column C	144.6 ?1.252 N=6
16	Difference between means	6.649 ?1.631
17	95% confidence interval	3.015 to 10.28
18	R squared	0.6243
19		
20	F test to compare variances	
21	F,DFn, Dfd	1.433, 5, 5
22	P value	0.7026
23	P value summary	ns
24	Are variances significantly different?	No

Figure 4

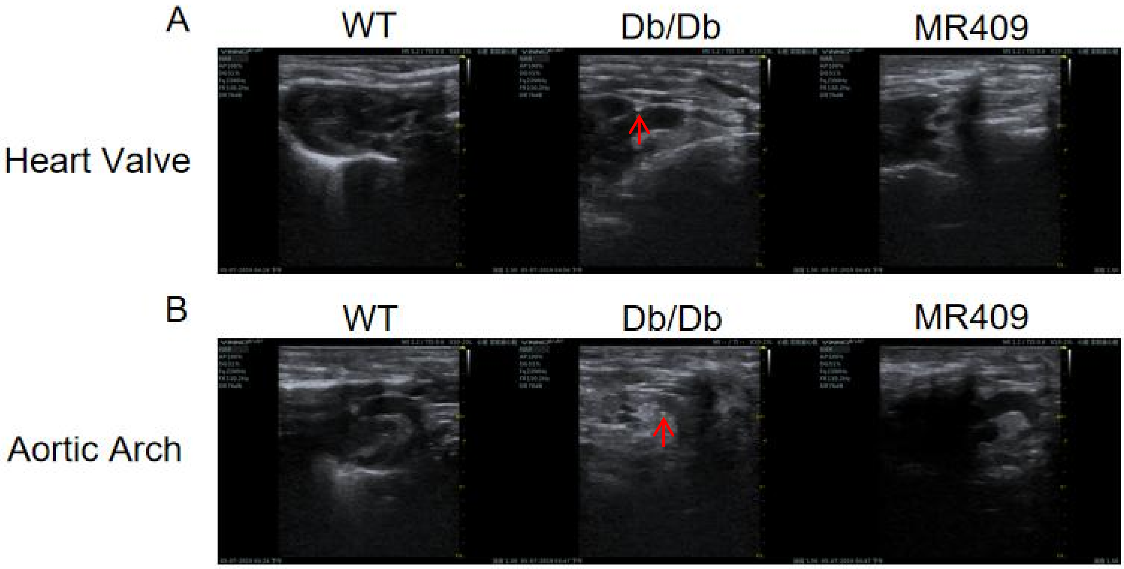

Figure 5

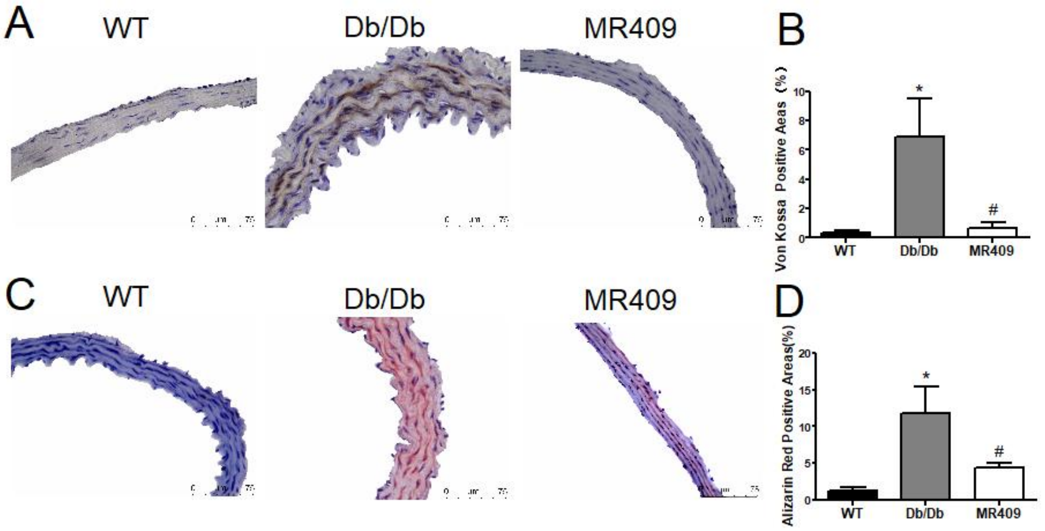

Fig 5A

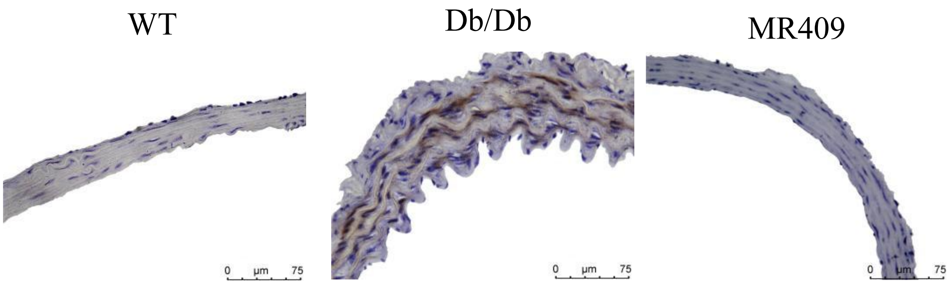

Fig 5B

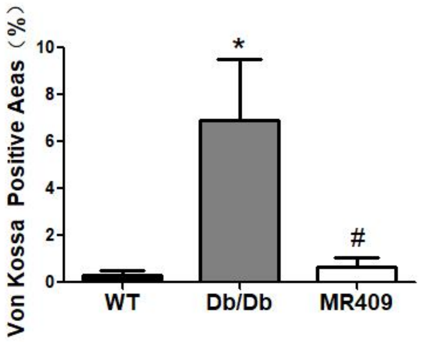

	A	B	C
	WT	Db/Db	MR409
	Y	Y	Y
1	0.261896	1.808272	2.537778
2	0.010528	4.234074	0.117041
3	0.477943	1.488847	0.259600
4	1.071609	4.398112	0.388364
5	0.047114	17.620730	0.378293
6	0.064090	11.754590	0.259600

1	Table Analyzed	Data 1
2	Column A	WT
3	vs	vs
4	Column B	Db/Db
5		
6	Unpaired t test	
7	P value	0.0159
8	P value summary	*
9	Are means signif. different? (P < 0.05)	Yes
10	One- or two-tailed P value?	One-tailed
11	t, df	t=2.493 df=10
12		
13	How big is the difference?	
14	Mean ± SEM of column A	0.3222 ?0.1662 N=6
15	Mean ± SEM of column B	6.884 ?2.627 N=6
16	Difference between means	-6.562 ?2.632
17	95% confidence interval	-12.43 to -0.6967
18	R squared	0.3832
19		
20	F test to compare variances	
21	F,DFn, Dfd	249.8, 5, 5
22	P value	< 0.0001
23	P value summary	***
24	Are variances significantly different?	Yes

1	Table Analyzed	Data 1
2	Column B	Db/Db
3	vs	vs
4	Column C	MR409
5		
6	Unpaired t test	
7	P value	0.0205
8	P value summary	*
9	Are means signif. different? (P < 0.05)	Yes
10	One- or two-tailed P value?	One-tailed
11	t, df	t=2.346 df=10
12		
13	How big is the difference?	
14	Mean ± SEM of column B	6.884 ?2.627 N=6
15	Mean ± SEM of column C	0.6568 ?0.3784 N=6
16	Difference between means	6.227 ?2.654
17	95% confidence interval	0.3134 to 12.14
18	R squared	0.3550
19		
20	F test to compare variances	
21	F,DFn, Dfd	48.22, 5, 5
22	P value	0.0006
23	P value summary	***
24	Are variances significantly different?	Yes

Fig 5C

WT Db/Db MR409

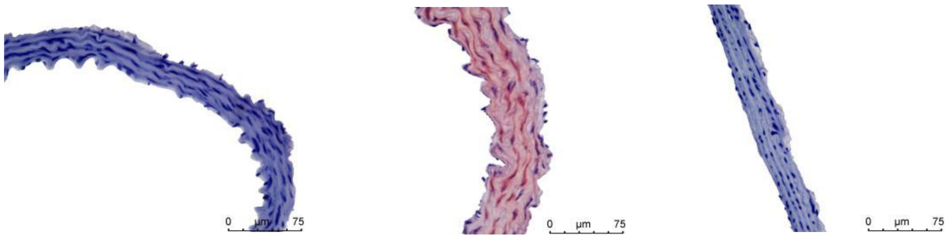

Fig 5D

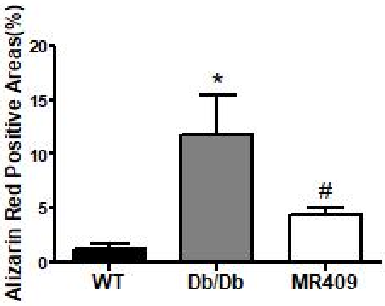

	A	B	C
	WT	Db/Db	MR409
	Y	Y	Y
1	2.364552	9.146991	3.661996
2	0.772449	5.801672	2.268403
3	2.217770	14.718350	3.542261
4	0.250602	6.018134	4.644092
5	0.847680	6.129132	6.463961
6	1.436284	28.847830	5.866663

1	Table Analyzed	Data 1
2	Column A	WT
3	vs	vs
4	Column B	Db/Db
5		
6	Unpaired t test	
7	P value	0.0090
8	P value summary	**
9	Are means signif. different? (P < 0.05)	Yes
10	One- or two-tailed P value?	One-tailed
11	t, df	t=2.825 df=10
12		
13	How big is the difference?	
14	Mean ± SEM of column A	1.315 ?0.3453 N=6
15	Mean ± SEM of column B	11.78 ?3.687 N=6
16	Difference between means	-10.46 ?3.703
17	95% confidence interval	-18.71 to -2.212
18	R squared	0.4439
19		
20	F test to compare variances	
21	F,DFn, Dfd	114.0, 5, 5
22	P value	< 0.0001
23	P value summary	***
24	Are variances significantly different?	Yes

1	Table Analyzed	Data 1
2	Column B	Db/Db
3	vs	vs
4	Column C	MR409
5		
6	Unpaired t test	
7	P value	0.0386
8	P value summary	*
9	Are means signif. different? (P < 0.05)	Yes
10	One- or two-tailed P value?	One-tailed
11	t, df	t=1.969 df=10
12		
13	How big is the difference?	
14	Mean ± SEM of column B	11.78 ?3.687 N=6
15	Mean ± SEM of column C	4.408 ?0.6402 N=6
16	Difference between means	7.369 ?3.742
17	95% confidence interval	-0.9677 to 15.71
18	R squared	0.2795
19		
20	F test to compare variances	
21	F,DFn, Dfd	33.17, 5, 5
22	P value	0.0015
23	P value summary	**
24	Are variances significantly different?	Yes

Figure 6

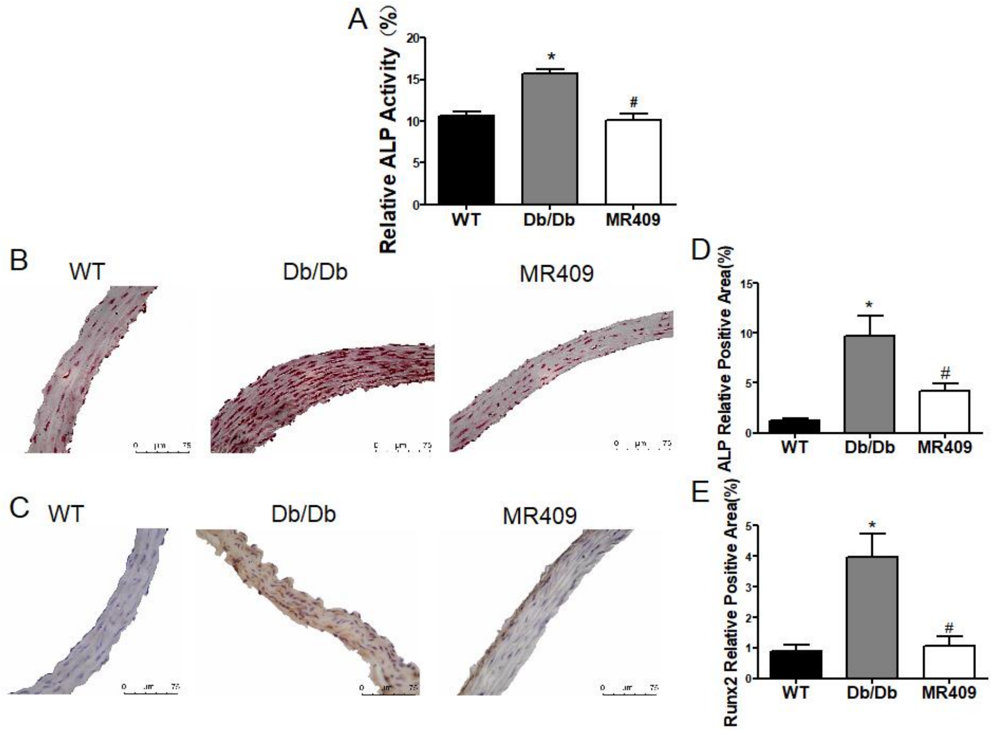

Fig 6A

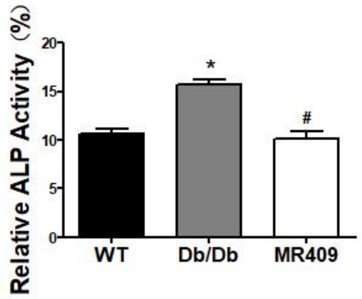

	A	B	C
	WT	Db/Db	MR409
	Y	Y	Y
1	11.250000	16.494250	10.597020
2	9.712644	13.505160	8.208093
3	9.381443	15.820900	12.408760
4	11.923080	16.878610	10.628570
5	9.364161	15.328470	10.862070
6	11.932770	16.571430	8.457143

1	Table Analyzed	Data 1
2	Column A	WT
3	vs	vs
4	Column B	Db/Db
5		
6	Unpaired t test	
7	P value	< 0.0001
8	P value summary	***
9	Are means signif. different? (P < 0.05)	Yes
10	One- or two-tailed P value?	One-tailed
11	t, df	t=7.203 df=10
12		
13	How big is the difference?	
14	Mean ± SEM of column A	10.59 ?0.5082 N=6
15	Mean ± SEM of column B	15.77 ?0.5074 N=6
16	Difference between means	-5.172 ?0.7181
17	95% confidence interval	-6.772 to -3.572
18	R squared	0.8384
19		
20	F test to compare variances	
21	F,DFn, Dfd	1.003, 5, 5
22	P value	0.9972
23	P value summary	ns
24	Are variances significantly different?	No

1	Table Analyzed	Data 1
2	Column B	Db/Db
3	vs	vs
4	Column C	MR409
5		
6	Unpaired t test	
7	P value	< 0.0001
8	P value summary	***
9	Are means signif. different? (P < 0.05)	Yes
10	One- or two-tailed P value?	One-tailed
11	t, df	t=6.760 df=10
12		
13	How big is the difference?	
14	Mean ± SEM of column B	15.77 ?0.5074 N=6
15	Mean ± SEM of column C	10.19 ?0.6497 N=6
16	Difference between means	5.573 ?0.8243
17	95% confidence interval	3.736 to 7.409
18	R squared	0.8205
19		
20	F test to compare variances	
21	F,DFn, Dfd	1.640, 5, 5
22	P value	0.6006
23	P value summary	ns
24	Are variances significantly different?	No

Fig 6B

WT Db/Db MR409

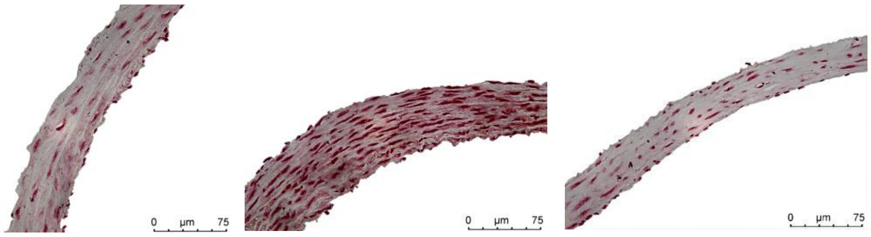

Fig 6C

WT Db/Db MR409

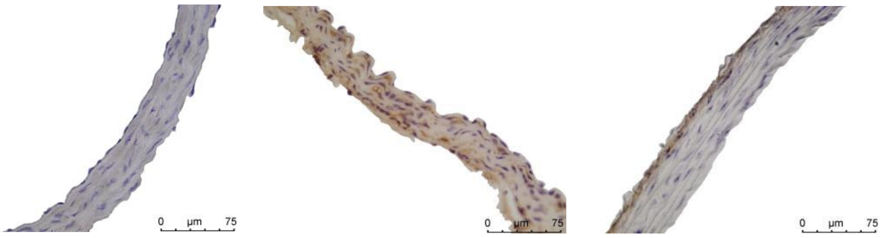

Fig 6D

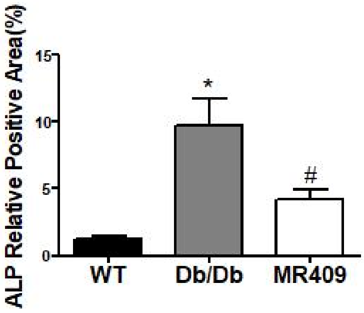

	A	B	C
	WT	Db/Db	MR409
	Y	Y	Y
1	1.053965	6.741989	4.562227
2	2.056440	5.419027	5.893169
3	0.965720	5.247566	4.266967
4	0.577913	18.322400	2.238351
5	1.689375	10.045460	2.104164
6	1.200096	12.428430	6.321914

1	Table Analyzed	Data 1
2	Column A	WT
3	vs	vs
4	Column B	Db/Db
5		
6	Unpaired t test	
7	P value	0.0012
8	P value summary	**
9	Are means signif. different? (P < 0.05)	Yes
10	One- or two-tailed P value?	One-tailed
11	t, df	t=4.053 df=10
12		
13	How big is the difference?	
14	Mean ± SEM of column A	1.257 ?0.2173 N=6
15	Mean ± SEM of column B	9.701 ?2.072 N=6
16	Difference between means	-8.444 ?2.083
17	95% confidence interval	-13.09 to -3.802
18	R squared	0.6216
19		
20	F test to compare variances	
21	F,DFn, Dfd	90.93, 5, 5
22	P value	0.0001
23	P value summary	***
24	Are variances significantly different?	Yes

1	Table Analyzed	Data 1
2	Column B	Db/Db
3	vs	vs
4	Column C	MR409
5		
6	Unpaired t test	
7	P value	0.0159
8	P value summary	*
9	Are means signif. different? (P < 0.05)	Yes
10	One- or two-tailed P value?	One-tailed
11	t, df	t=2.492 df=10
12		
13	How big is the difference?	
14	Mean ± SEM of column B	9.701 ?2.072 N=6
15	Mean ± SEM of column C	4.231 ?0.7243 N=6
16	Difference between means	5.470 ?2.195
17	95% confidence interval	0.5791 to 10.36
18	R squared	0.3831
19		
20	F test to compare variances	
21	F,DFn, Dfd	8.184, 5, 5
22	P value	0.0377
23	P value summary	*
24	Are variances significantly different?	Yes

Fig 6E

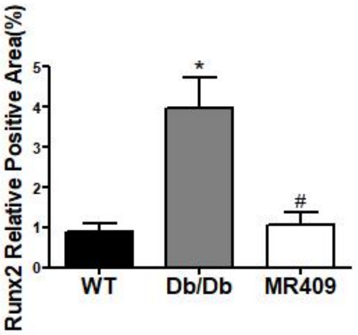

	A	B	C
	WT	Db/Db	MR409
	Y	Y	Y
1	0.746440	3.776573	0.358438
2	0.391043	4.688545	0.920549
3	0.566168	5.811803	2.512895
4	1.915324	3.776573	1.011707
5	0.945503	0.545089	0.885460
6	0.873933	5.271431	0.732852

1	Table Analyzed	Data 1
2	Column A	WT
3	vs	vs
4	Column B	Db/Db
5		
6	Unpaired t test	
7	P value	0.0015
8	P value summary	**
9	Are means signif. different? (P < 0.05)	Yes
10	One- or two-tailed P value?	One-tailed
11	t, df	t=3.877 df=10
12		
13	How big is the difference?	
14	Mean ± SEM of column A	0.9064 ?0.2181 N=6
15	Mean ± SEM of column B	3.978 ?0.7617 N=6
16	Difference between means	-3.072 ?0.7923
17	95% confidence interval	-4.837 to -1.307
18	R squared	0.6005
19		
20	F test to compare variances	
21	F,DFn, Dfd	12.19, 5, 5
22	P value	0.0158
23	P value summary	*
24	Are variances significantly different?	Yes

1	Table Analyzed	Data 1
2	Column B	Db/Db
3	vs	vs
4	Column C	MR409
5		
6	Unpaired t test	
7	P value	0.0026
8	P value summary	**
9	Are means signif. different? (P < 0.05)	Yes
10	One- or two-tailed P value?	One-tailed
11	t, df	t=3.547 df=10
12		
13	How big is the difference?	
14	Mean ± SEM of column B	3.978 ?0.7617 N=6
15	Mean ± SEM of column C	1.070 ?0.3034 N=6
16	Difference between means	2.908 ?0.8199
17	95% confidence interval	1.081 to 4.735
18	R squared	0.5571
19		
20	F test to compare variances	
21	F,DFn, Dfd	6.303, 5, 5
22	P value	0.0646
23	P value summary	ns
24	Are variances significantly different?	No

Figure7

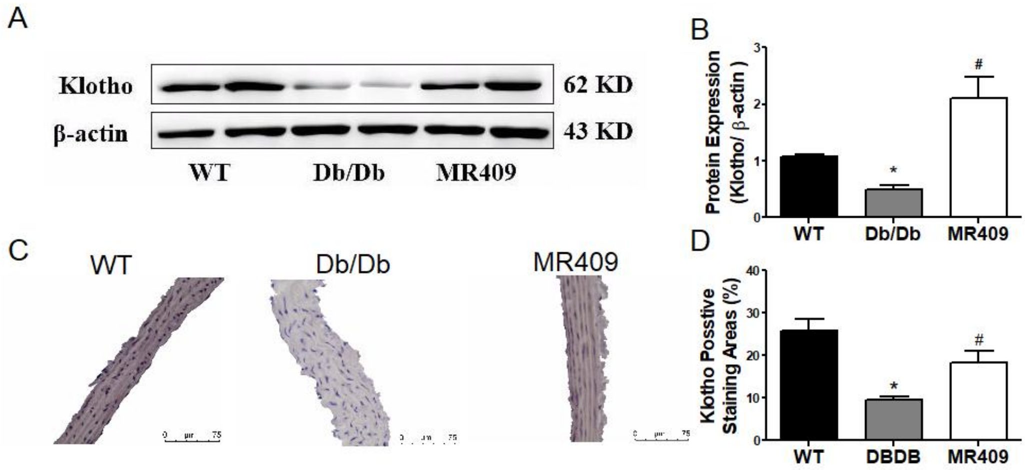

Fig 7A

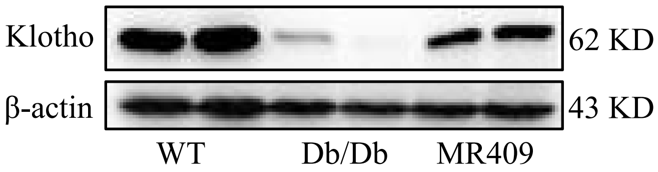

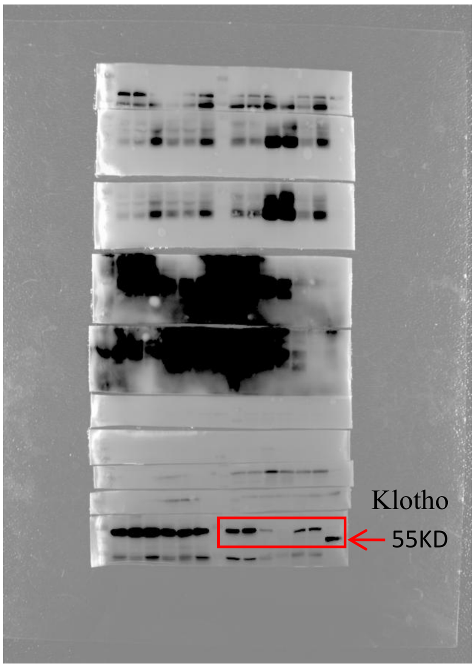

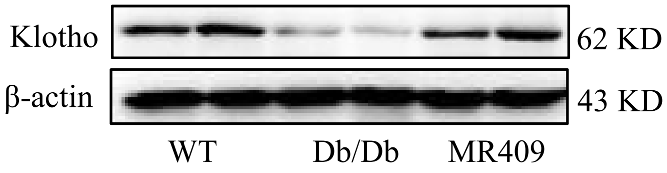

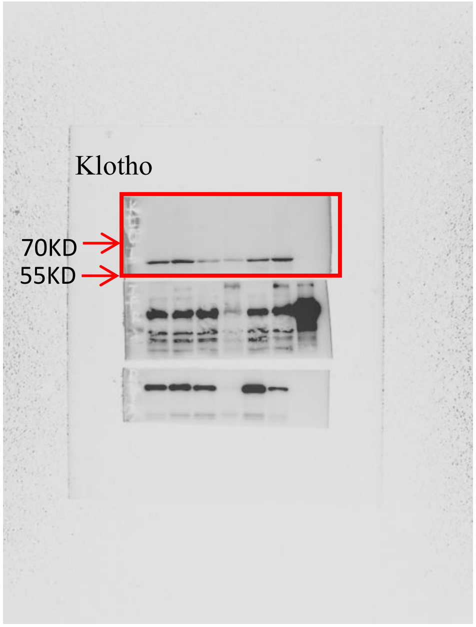

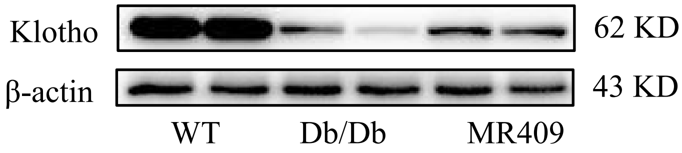
Klotho
62 KD
β-actin
43 KD
WT
Db/Db
MR409

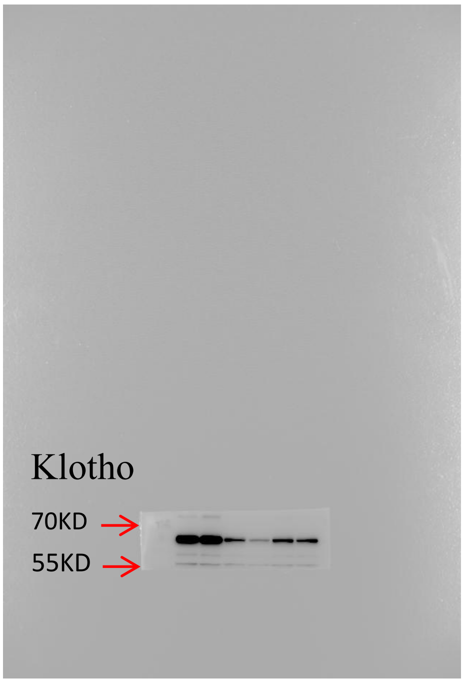
Klotho
70KD
55KD

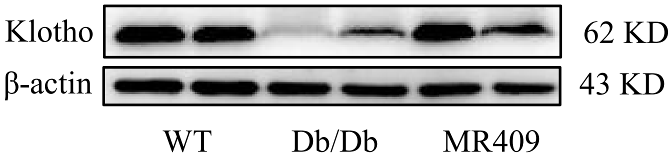
Klotho
62 KD
β-actin
43 KD
WT
Db/Db
MR409

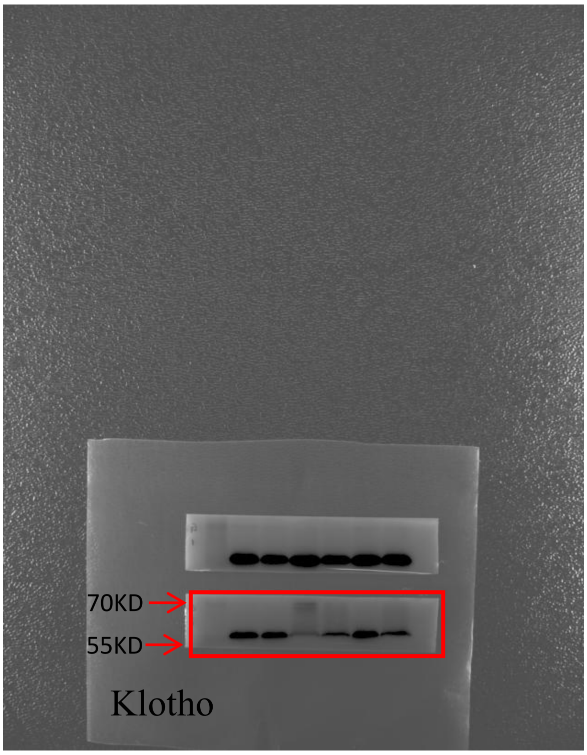
70KD
55KD
Klotho

Fig 7B

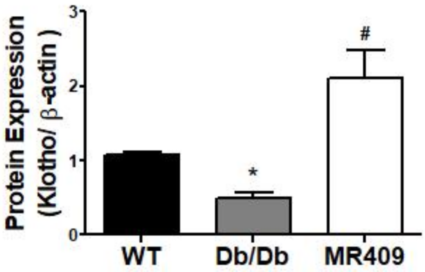

	A	B	C
	WT	Db/Db	MR409
	Y	Y	Y
1	1.000000	0.277088	2.771730
2	1.218837	0.697957	1.367257
3	1.000000	0.282530	2.824638
4	1.225143	0.672887	1.389941
5	1.000000	0.334537	3.186718
6	1.008879	0.661300	1.108892

	t test	
1	Table Analyzed	Data 1
2	Column A	WT
3	vs	vs
4	Column B	Db/Db
5		
6	Unpaired t test	
7	P value	< 0.0001
8	P value summary	***
9	Are means signif. different? (P < 0.05)	Yes
10	One- or two-tailed P value?	One-tailed
11	t, df	t=6.051 df=10
12		
13	How big is the difference?	
14	Mean ± SEM of column A	1.075 ?0.04636 N=6
15	Mean ± SEM of column B	0.4877 ?0.08535 N=6
16	Difference between means	0.5878 ?0.09713
17	95% confidence interval	0.3714 to 0.8042
18	R squared	0.7855
19		
20	F test to compare variances	
21	F,DFn, Dfd	3.390, 5, 5
22	P value	0.2065
23	P value summary	ns
24	Are variances significantly different?	No

1	Table Analyzed	Data 1
2	Column B	Db/Db
3	vs	vs
4	Column C	MR409
5		
6	Unpaired t test	
7	P value	0.0009
8	P value summary	***
9	Are means signif. different? (P < 0.05)	Yes
10	One- or two-tailed P value?	One-tailed
11	t, df	t=4.232 df=10
12		
13	How big is the difference?	
14	Mean ± SEM of column B	0.4877 ?0.08535 N=6
15	Mean ± SEM of column C	2.108 ?0.3733 N=6
16	Difference between means	-1.620 ?0.3829
17	95% confidence interval	-2.474 to -0.7673
18	R squared	0.6417
19		
20	F test to compare variances	
21	F,DFn, Dfd	19.13, 5, 5
22	P value	0.0057
23	P value summary	**
24	Are variances significantly different?	Yes

Fig 7C

WT Db/Db MR409

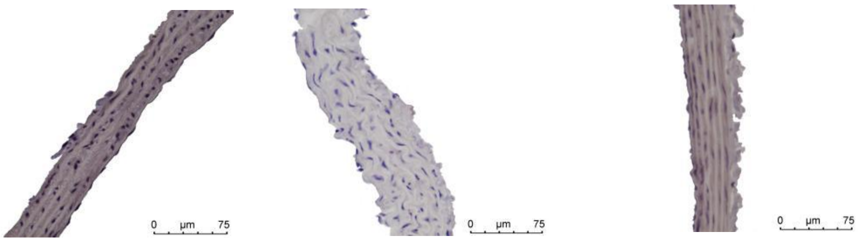

Fig 7D

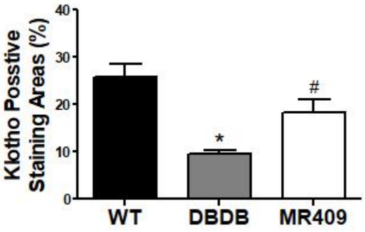

	A	B	C
	WT	DBDB	MR409
	Y	Y	Y
1	0.232181	0.125280	0.140077
2	0.188976	0.079362	0.215937
3	0.356031	0.096114	0.090878
4	0.196890	0.093869	0.202991
5	0.248465	0.101240	0.294122
6	0.326753	0.084538	0.152167

1	Table Analyzed	Data 1
2	Column A	WT
3	vs	vs
4	Column B	DBDB
5		
6	Unpaired t test	
7	P value	0.0001
8	P value summary	***
9	Are means signif. different? (P < 0.05)	Yes
10	One- or two-tailed P value?	One-tailed
11	t, df	t=5.607 df=10
12		
13	How big is the difference?	
14	Mean ± SEM of column A	0.2582 ?0.02804 N=6
15	Mean ± SEM of column B	0.09673 ?0.006568 N=6
16	Difference between means	0.1615 ?0.02880
17	95% confidence interval	0.09731 to 0.2257
18	R squared	0.7586
19		
20	F test to compare variances	
21	F,DFn, Dfd	18.23, 5, 5
22	P value	0.0063
23	P value summary	**
24	Are variances significantly different?	Yes

1	Table Analyzed	Data 1
2	Column B	DBDB
3	vs	vs
4	Column C	MR409
5		
6	Unpaired t test	
7	P value	0.0080
8	P value summary	**
9	Are means signif. different? (P < 0.05)	Yes
10	One- or two-tailed P value?	One-tailed
11	t, df	t=2.897 df=10
12		
13	How big is the difference?	
14	Mean ± SEM of column B	0.09673 ?0.006568 N=6
15	Mean ± SEM of column C	0.1827 ?0.02893 N=6
16	Difference between means	-0.08596 ?0.02967
17	95% confidence interval	-0.1521 to -0.01986
18	R squared	0.4564
19		
20	F test to compare variances	
21	F,DFn, Dfd	19.40, 5, 5
22	P value	0.0055
23	P value summary	**
24	Are variances significantly different?	Yes
